# Systems Analysis of the Dynamic Inflammatory Response to Tissue Damage Reveals Spatiotemporal Properties of the Wound Attractant Gradient

**DOI:** 10.1016/j.cub.2016.06.012

**Published:** 2016-08-08

**Authors:** Helen Weavers, Juliane Liepe, Aaron Sim, Will Wood, Paul Martin, Michael P.H. Stumpf

**Affiliations:** 1 Department of Biochemistry, School of Medical Sciences, University of Bristol, Bristol BS8 1TD, UK; 2 Theoretical Systems Biology, Division of Molecular Biosciences, Imperial College London, London SW7 2AZ, UK; 3 School of Cellular and Molecular Medicine, Medical Sciences, University of Bristol, Bristol BS8 1TD, UK; 4 Department of Physiology, Pharmacology and Neuroscience, Faculty of Biomedical Sciences, University of Bristol, Bristol BS8 1TD, UK; 5 School of Medicine, Cardiff University, Cardiff CF14 4XN, UK; 6 Lee Kong Chian School of Medicine, Nanyang Technologicial University, Singapore 636921, Singapore

## Abstract

In the acute inflammatory phase following tissue damage, cells of the innate immune system are rapidly recruited to sites of injury by pro-inflammatory mediators released at the wound site. Although advances in live imaging allow us to directly visualize this process in vivo, the precise identity and properties of the primary immune damage attractants remain unclear, as it is currently impossible to directly observe and accurately measure these signals in tissues. Here, we demonstrate that detailed information about the attractant signals can be extracted directly from the in vivo behavior of the responding immune cells. By applying inference-based computational approaches to analyze the in vivo dynamics of the *Drosophila* inflammatory response, we gain new detailed insight into the spatiotemporal properties of the attractant gradient. In particular, we show that the wound attractant is released by wound margin cells, rather than by the wounded tissue per se, and that it diffuses away from this source at rates far slower than those of previously implicated signals such as H_2_O_2_ and ATP, ruling out these fast mediators as the primary chemoattractant. We then predict, and experimentally test, how competing attractant signals might interact in space and time to regulate multi-step cell navigation in the complex environment of a healing wound, revealing a period of receptor desensitization after initial exposure to the damage attractant. Extending our analysis to model much larger wounds, we uncover a dynamic behavioral change in the responding immune cells in vivo that is prognostic of whether a wound will subsequently heal or not.

**Video Abstract:**

## Highlights

• Computational modeling of in vivo inflammatory response to tissue damage is applied
• The model infers novel spatiotemporal properties of the wound attractant gradient
• Wound signal is released from the wound edge for 30 min and diffuses at 200 μm^2^/min
• Modeling two competing wounds reveals a period of immune cell desensitization

## Graphical Abstract

**Figure:**
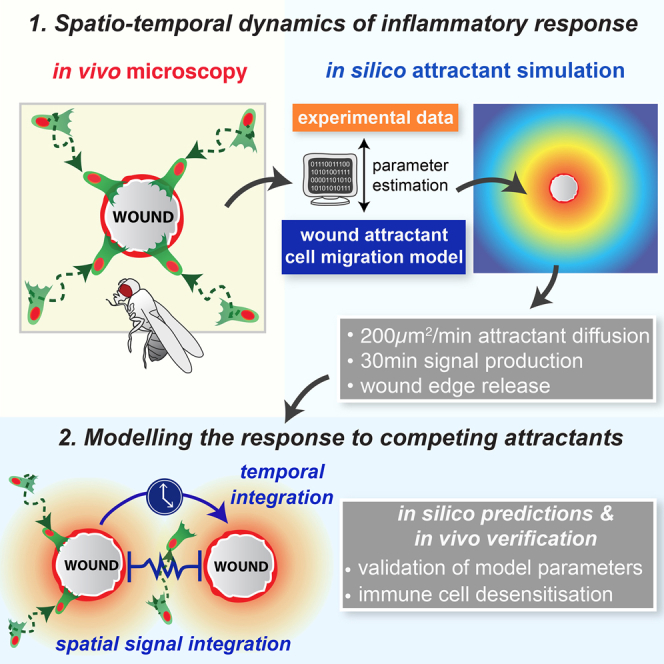

## Teaser

Weavers et al. use Bayesian modeling of the inflammatory response to tissue damage in *Drosophila* to extract novel spatiotemporal properties of the wound attractant, including its diffusion coefficient, duration, and source of signal production. Modeling the response to two competing attractants uncovers a period of immune cell desensitization.

## Introduction

In the acute inflammatory response to tissue damage, cells of the innate immune system are rapidly drawn to the injury site, where they perform a number of essential functions, including killing invading pathogens, clearing damaged necrotic tissue, promoting matrix deposition, and tissue remodeling [1]. Recent advances in imaging technology now enable us to observe this inflammatory response at high resolution in vivo and obtain a detailed time course for leukocyte infiltration [2, 3]. However, one of the major remaining challenges is identifying the danger signals released upon tissue wounding that set up the chemotactic gradients that draw migrating leukocytes into the injury site—and, moreover, determining precisely how these different chemoattractant signals propagate through, and interact within, the tissue to control the complex spatiotemporal dynamics of inflammatory cell recruitment.

Genetic approaches have implicated a number of damage signals (damage-associated molecular patterns; DAMPs) in facilitating efficient cell recruitment into the injury site. These include extracellular ATP, mitochondrial formylated peptides, and mitochondrial DNA, which are detected by pattern recognition receptors (PRRs) located on the surface of innate immune cells following their release from damaged cells [4, 5, 6]. Reactive oxygen species (ROS) are also important players, with genetic studies in zebrafish and *Drosophila* indicating that efficient leukocyte migration to wounds is dependent on the release of hydrogen peroxide (H_2_O_2_) from the injury site [7, 8, 9, 10, 11].

Nevertheless, it is currently impossible to directly observe and accurately quantify these implicated attractant signals, due to the limited and inadequate reporter tools that are available at this time. Since we cannot directly visualize their dynamic distribution within the damaged tissue, it is unclear whether they function as true pro-inflammatory chemoattractants, generating a functional chemotactic gradient that draws leukocytes into the injury site, or, alternatively, whether these signals potentiate the leukocyte response to another, as yet unknown, chemoattractant. It is also becoming clear that, in the complex environment of a healing wound, immune cells will encounter a large network of different inflammatory mediators [4, 12]. We must, therefore, not only understand the behavior of individual attractants but also learn how competing (and overlapping) inflammatory signals interact in space and time to regulate more complex immune cell behavior [13].

Although the acute inflammatory response is an inevitable outcome of any tissue damage, it is of immense clinical relevance because an inappropriate inflammatory response is often associated with, and potentially responsible for, a wide range of pathologies, including chronic non-healing wounds, excessive scarring, and predisposition to cancer [14, 15]. A more detailed understanding of the inflammatory signals orchestrating this response is, therefore, key to the development of new prognostic indicators and strategies for the treatment of chronic inflammatory diseases, which might protect healing tissue from excessive and unbalanced inflammation.

Over recent decades, *Drosophila* has become an attractive model system in which to study the wound inflammatory response [16]. *Drosophila* embryos and pupae offer optical translucency to enable unparalleled, high-resolution in vivo imaging and genetic tractability that is greater than other models (such as mice or zebrafish) currently offer. We and others have demonstrated that sterile injury to the *Drosophila* epithelium results in rapid and robust recruitment of innate immune cells (hemocytes) to the site of damage, establishing *Drosophila* as a valuable model to elucidate important features of the immune response conserved from insects to man [10, 17, 18, 19].

Since the signaling gradient that draws leukocytes toward the site of injury cannot be directly measured experimentally, we require novel computational approaches to, instead, extract this information from the behavioral response of innate immune cells. Until now, analysis of immune cell recruitment has mainly focused on simple statistics, e.g., counting numbers of recruited cells, cell velocity, and the straightness index, which give only basic insights into cell behavior. However, such simple statistics fail to capture the full behavioral information content offered by such rich in vivo imaging data. For this reason, we have developed more sophisticated computational tools, based on commonly used random walk models in biology [20, 21], that allow us to elucidate signaling gradient characteristics from patterns of leukocyte behavior in vivo [22].

In this study, we develop these automated tracking and computational approaches to analyze the spatiotemporal behavior of innate immune cells following injury in *Drosophila*. We extract the immune cell trajectories from in vivo time-lapse imaging data to first compute how cell directionality changes upon wounding, and then we apply inference-based statistical methods to reveal new details of the wound-induced chemoattractant(s). In this way, we quantify the chemotactic gradient responsible for immune cell attraction to wounds, inferring parameters such as its diffusion coefficient, its source, and the duration of active signal production, and we reveal precisely how the immediate damage signal spreads out from the injury site in space and time. Extrapolating these data to more complex scenarios, we generate testable predictions about leukocyte behavior that will arise in the presence of competing attractant signals. Finally, by extending our analysis to large non-healing wounds, we begin to dissect the complex signaling processes responsible for pathological chronic inflammation and reveal how dynamic immune cell behavior is a novel prognostic indicator of chronic wounds. Our study highlights the valuable insight that can be extracted from in vivo imaging data, if more sophisticated analysis tools are used, that would otherwise remain experimentally inaccessible.

## Results

### High-Resolution In Vivo Imaging and Automated 3D Tracking of the *Drosophila* Acute Inflammatory Response

**Figure 1:**
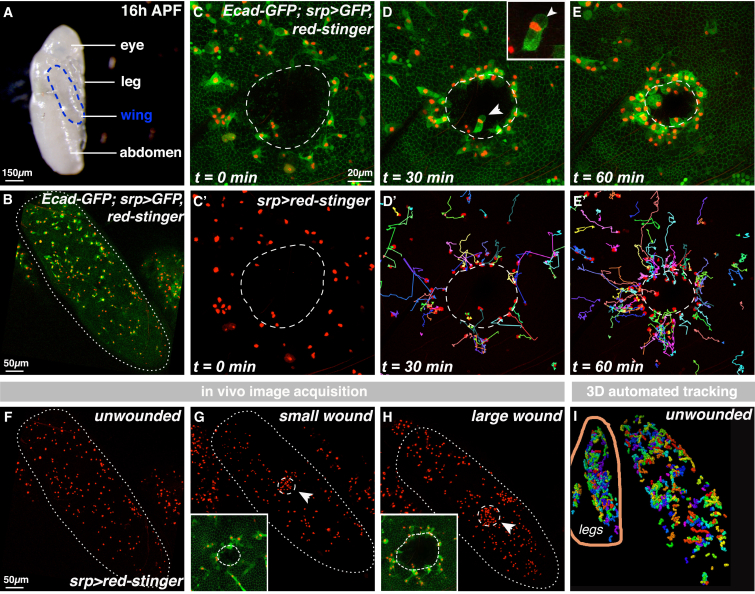
In Vivo Imaging and 3D Tracking of the Acute Inflammatory Response (A–E′) The pupal wing (outlined in A and B) contains innate immune cells (hemocytes, B; *srp-Gal4* drives hemocyte expression of *UAS-nuclear-red-stinger* and *UAS-GFP*). Upon wounding the epithelium (dashed outline, C and C′; *Ecadherin-GFP*), hemocytes are recruited toward the damage site (D and E; multicolored cell tracks in D′ and E′), where they phagocytose wound debris (phagocytic vacuole indicated by arrows in D and inset). (F–H) in vivo data acquisition: unwounded controls for basal hemocyte behavior (F) and two wound sizes for the spatiotemporal dynamics of hemocyte wound recruitment (arrows and insets; small, 55 μm, wounds in G and large, 110 μm, wounds in H). (I) 3D automated tracking extracts cell trajectories (shown for control unwounded wings). Data from outside the wing (e.g., legs) were manually excluded. See also Figure S1 and Movies S1 and S2.

We have exploited the optical transparency and genetic tractability of the pupal stage of *Drosophila* development to follow the in vivo behavior of innate immune cells (hemocytes) in response to sterile tissue damage (Figure 1) [19]. Although we have previously used the *Drosophila* embryo to image the inflammatory response [9, 10, 17, 18], here, we have developed a pupal wing model because it offers some distinct advantages for imaging. Wounded pupae can be imaged over longer time periods, more tissue space is available for experimental perturbation, and there are significantly more hemocytes at this stage, providing more cell trajectories for improved statistical power during later mathematical analysis. As with embryos and larvae [17, 23], although these wounds exhibit a robust inflammatory response, deletion of all immune cells (by expressing the pro-apoptotic gene *reaper* specifically in immune cells) does not hinder the re-epithelialization process (Figures S1A–S1J).

Pupae were removed from their protective pupal cases (Figure 1A) and imaged using confocal time-lapse microscopy (Figures 1B–1E). At this stage in pupal development, 16–20 hr APF (after puparium formation), the pupal wing (Figure 1B) is a simple bilayered structure consisting of two large flat epithelial sheets connected around the wing periphery [24]. Hemolymph (insect blood) occupies the space between these two epithelial layers and contains large numbers of migratory hemocytes (red nuclei; Figure 1B). Injury to the pupal wing epithelium induced a rapid and robust migration of hemocytes to the wound site (Figures 1C–1E; Movie S1). Manual tracking of hemocyte trajectories highlighted the complex spatiotemporal dynamics of this response (Figures 1C′–1E′; multicolored tracks), similar to that reported previously for wounded embryos [17, 18].

In order to comprehensively analyze the spatiotemporal wound recruitment of hemocytes, we developed automated 3D cell-tracking software and generated large in vivo imaging datasets of hemocyte behavior, recorded with high temporal and spatial resolution, using the GAL4-UAS system [25] to drive hemocyte-specific expression of nuclear “red stinger” RFP (red fluorescent protein; a fast-maturing variant of DsRed with a C-terminal nuclear localization signal [26]) to enable nuclear tracking (Figures 1F–1H; Movie S2), which correlates well with dynamic cell directionality (Figures S1K and S1L). Three different experimental conditions were compared: naive unwounded tissue gave information on basal hemocyte behavior (Figure 1F), while acute inflammatory data were obtained for two different wound sizes (small 55 μm- and large 110 μm-diameter wounds) (Figures 1G and 1H). 3D automated nuclear tracking mapped all hemocyte trajectories for each of the three conditions; we manually excluded any data located outside of the wing (e.g., from pupal legs; control unwounded image in Figure 1I). These trajectory data were then statistically interrogated to provide detailed analysis of hemocyte behavior.

### Calibration of Random Walk Models to In Vivo Immune Trajectories Reveals a Strong Spatiotemporal Dependence of Hemocyte Wound Recruitment

**Figure 2:**
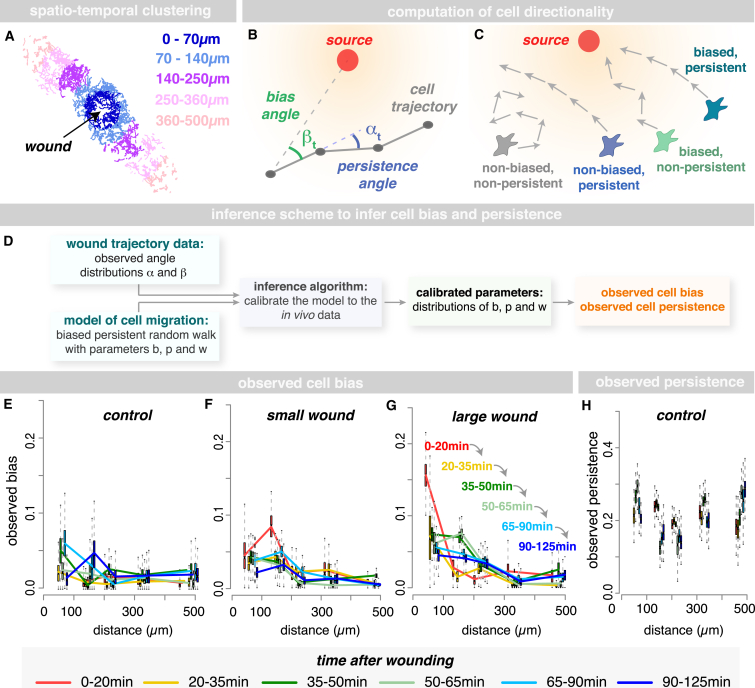
Modeling Spatiotemporal Dynamics of the Inflammatory Response (A–D) Trajectories are subdivided into five spatial clusters (A). Cell directionality inferred using a biased persistent random walk model (B and C) and trajectories described as a sequence of motion vectors (gray, B) between consecutive time points (black dots, B). Cell bias and persistence are inferred from the angles β_t_ (green, B) between the motion vector and the direction toward the source (red dot, B) and the angles α_t_ (blue, B) between the current and preceding motion vector, using an inference-based approach (D, with b and p describing the persistence and bias parameters, respectively, and w describing the probability of a biased motion). (E–H) Control hemocytes migrate with a constant persistence (H) and very low basal bias (E). Injury causes a rapid increase in bias toward the wound (small wound, F; large wound, G); cells located nearest to the injury (0–100 μm) respond most rapidly (red lines, F and G). Hemocytes distant to the wound respond at successively later time points (yellow, green, and blue lines in G). Boxplots represent estimated marginal posterior parameter distributions for observed bias (E–G) and persistence (H), showing the full distribution with median and 5^th^, 25^th^, 75^th^, and 95^th^ percentiles. See also Figure S2.

To gain detailed information about the spatiotemporal changes in hemocyte behavior in response to tissue damage, we grouped the extracted cell trajectories according to both distance and time post-wounding (Figure 2A), giving five different temporal clusters (T1–T5) and five spatial clusters (S1–S5; Figure 2A). Hemocyte behavior is quantified using a biased persistent random walk model, which is commonly used to describe leukocyte migration [21]. Here, the overall movement of a cell depends on both its directional bias and its persistence (Figures 2B and 2C). Directional bias is a measure of how leukocyte movement is oriented toward an external stimulus (Figure 2B, green), while directional persistence is a measure of a cell’s tendency to keep moving in the same direction (Figure 2B, blue). In this way, we have determined precisely how hemocyte directionality changes upon wounding.

We described the hemocyte movement as a sequence of angles γ_t_ (motion vectors; gray tracks in Figure 2B) between consecutive time points t and t + 1 (Figure 2B). For each motion vector, we computed two characteristics: (1) the angle β_t_ between the motion vector γ_t_ and the direction toward the wound (Figure 2B) and (2) the angle α_t_ between the motion vector γ_t_ and the preceding motion vector γ_t − 1_ (Figure 2B). The distribution of the angles β indicates the bias of a cell’s migration toward the wound, while the distribution of the angles α provides information about the persistence of that cell’s migration. These angle distributions are then used to infer the observed cell bias and persistence in each spatiotemporal cluster following wounding on a scale ranging from 0 to 1, with 0 indicating zero bias (or persistence) and 1 indicating maximal bias (or persistence). This was performed using a “Bayesian inference-based approach” (Figure 2D; for details, see Experimental Procedures and Supplemental Methodological Primer in the Supplemental Information), in which we calibrated our model of cell migration to fit the angle distributions of α and β obtained from trajectory data.

In unwounded controls, hemocytes migrate with a constant level of persistence and with a very low basal bias, as expected (Figures 2E and 2H). Upon wounding, there is an immediate and marked increase in hemocyte bias toward the injury site, with cells located closest to the injury (0–100 μm) responding most rapidly and showing the largest increase in cell bias during the first 20 min following injury (Figures 2F and 2G, red lines). Hemocytes located progressively further away from the injury site respond to the wound at successively later time points, as shown by their respective increases in cell bias (yellow, green, and blue lines, respectively; Figure 2G). In this way, a single “wave” of cell responsiveness can be seen spreading outward from the wound (Figure 2G, arrows), which we envision to reflect the diffusion of the wound chemoattractant(s) away from the injury site, and this provides a starting point from which to infer specific properties of the wound attractant (see below). Surprisingly, wounding only induced a change in cell bias and did not cause a detectable change in the persistence of the migrating immune cells (Figures S2A and S2B).

We ruled out the possibility that the observed hemocyte movement toward the wound site is driven, in part, by bulk tissue flow by co-injection of inert beads injected into the extracellular space (Figures S1M–S1R). Bead movement within the hemolymph did not correlate at all with hemocyte migration in unwounded (Figures S1M and S1N) or wounded (Figures S1O–S1Q) pupae, except when beads were phagocytosed by hemocytes (Figure S1Q); beads engulfed by epithelial cells exhibited little movement (Figure S1R).

We tested whether immune cells might interact and influence one another’s trajectories by contact inhibition of locomotion (CIL), as occurs during developmental dispersal of these cells [27]. In silico simulations where such contact inhibition behaviors occurred during the wound inflammatory response predict the recruitment of far fewer immune cells than we see in our in vivo experiments (Figures S2C–S2F), suggesting that CIL does not occur at the wound site. Indeed, high-magnification imaging of immune cells as they contact one another close to the wound confirms that CIL rules do not apply during the wound response (Figures 1C–1E). It will be interesting to determine how the CIL machinery is shut down at wound sites and whether this is universal across other inflammation scenarios.

### Bayesian Inference Quantifies the Wound-Induced Chemoattractant Gradient

**Figure 3:**
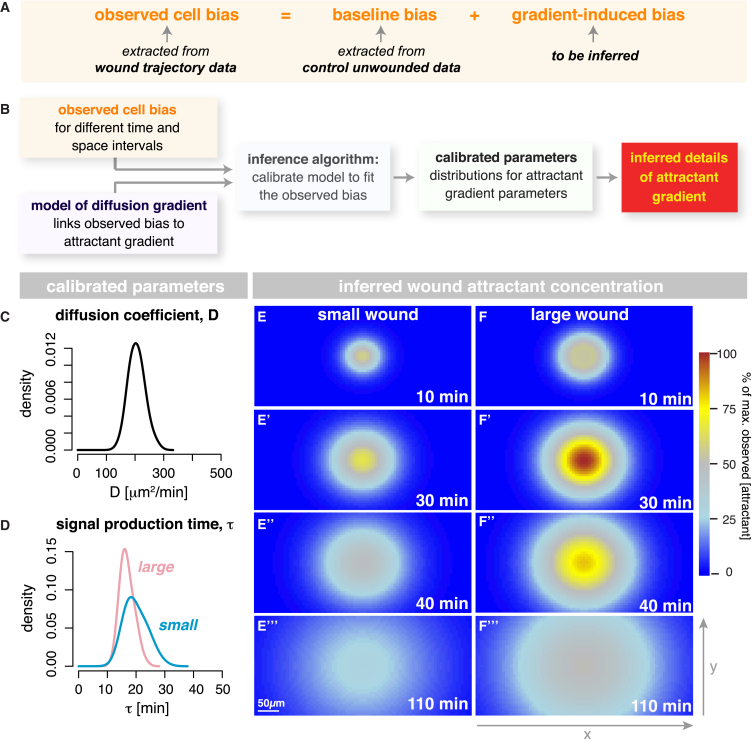
Quantification of the Wound-Induced Chemoattractant Gradient (A and B) Cell bias depends on “baseline bias,” which exists in the absence of injury, and “wound-induced bias,” triggered by tissue damage (A). In (B), a wound attractant gradient is modeled, using a standard 2D diffusion model. Using a Bayesian inference approach, we infer the set of parameters that best explains our experimental data. (C–F′′′) The best-fit model indicates that the wound attractant diffuses at approximately 200 μm^2^/min (C) and is actively produced by the wound for ∼30 min (D). Quantification of the spatiotemporal behavior of the wound attractant for both small (E–E′′′) and large (F–F′′′) wounds (heatmaps in E and F). Colors (see scale bar) represent attractant concentrations relative to the highest predicted concentration from 0% to 100%. See also Figure S3 and Movie S3.

Our computational model describes the wound attractant gradient and links its dynamics to the observed hemocyte directional bias. This enabled us to extract detailed characteristics of the wound attractant from our hemocyte trajectory data (Figure 3). In simple terms, the bias exhibited by a migrating hemocyte depends on (1) the level of baseline bias that exists before tissue damage (e.g., due to any tissue constraints) and (2) the additional bias triggered by wounding (Figure 3A). Wound-induced bias is a function of both the attractant gradient and attractant receptor-ligand binding kinetics. We describe the attractant gradient using a standard 2D diffusion model, which includes parameters for the total attractant source strength, the duration of active signal production at the source, and the signal diffusion rate (see Experimental Procedures for details).

Hypothetically, the wound signal could either be released from the damaged tissue occupying the entire wound area or, alternatively, by the marginal epithelial cells around the wound circumference. We initially modeled both scenarios, resulting in two different attractant gradient models (see Experimental Procedures for details of the model) and then determined which model best represents our experimental trajectory data. To do this, we applied a Bayesian inference approach, similar to the one described in the previous section, using the information gathered about the hemocytes’ directional bias in response to small versus large wounds (Figure 3B). We found that the best model is that in which the wound attractant emanates from the wound margin (Figures S3A–S3D). The total signaling strength of the wound (i.e., total flow of attractant from source) increases in proportion with wound circumference rather than area. Since there appears to be only one temporal response to the wound, and this is proportional with increasing wound circumference, we suggest that the source of the signal is most likely to be the epithelial margin rather than the damaged wound area.

The analysis of the parameter distributions from this best-fit model indicates that the chemoattractant released upon wounding diffuses away from the site of tissue damage at a rate of approximately 200μm^2^/min (Figure 3C). Our data indicate that the new chemoattractant signal is actively produced by the wound until ∼30 min after the initial injury (Figure 3D); after this time, the chemoattractant will continue diffusing away from the wound at 200 μm^2^/min, but no new signal is released. Interestingly, the duration of active signal production in large wounds is similar to that for small wounds (Figure 3D), suggesting that large wounds will stop actively releasing new chemoattractant long before they have finished closing. Our data, therefore, allow us to quantify the spatiotemporal behavior of the wound chemoattractant for the first time (heatmaps in Figures 3E and 3F; Figures S3E and S3F; Movie S3). From these data, we found no evidence to support the existence of a second wave of “cell responsiveness” (or change in bias toward the wound) suggesting that there is only one wave of wound signal, although this does not entirely rule out the possibility of there being multiple attractants released by the wound at the same time.

Our data suggest that the wound attractant diffuses at rates slower than those of previously implicated signals such as ATP [28, 29]. Indeed, we found that hemocyte-specific knockdown of the *Drosophila* ATP receptor (Ado-R) does not affect hemocyte responsiveness to a wound (Figures S3G–S3I), suggesting that ATP is not involved in hemocyte attraction to wounds. This is also consistent with previous work from our lab [9] demonstrating that *ado-R* null mutant embryos exhibit a completely normal inflammatory response to wounding.

### Predictive Modeling of Immune Cell Behavior in Complex Fields of Competing Attractants

Thus far, we have modeled relatively simple scenarios involving the pro-inflammatory response to a single chemoattractant released from individual wounds. However, wounds are often far more complex, and immune cells must navigate through fields of multiple overlapping attractant cues [4, 12, 13]. Taking advantage of the insights gained from our modeling analysis, we used the attractant data, extracted from our single-wound studies, to predict hemocyte behavior in more complex environments; for example, in the presence of two or more competing attractant sources. By subsequently testing these predictions experimentally in vivo, we have further validated our cell migration and attractant gradient models (including the estimated attractant parameters).

**Figure 4:**
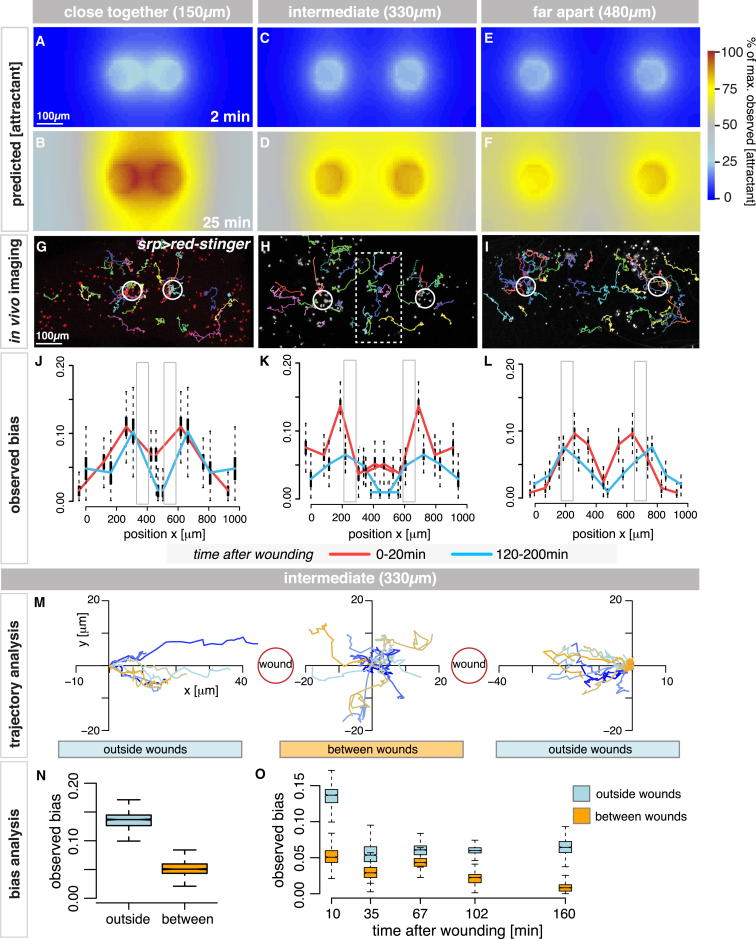
Modeling the Inflammatory Response to Competing Attractant Cues (A–F) For wounds close together, attractant gradients overlap and mimic a single, very large wound (A and B), predicting less biased migration in the inter-wound region. For wounds far apart, attractant gradients will not interact (E and F), and hemocytes respond as for two single wounds. For wounds of intermediate distance apart (C), attractant gradients will strongly overlap by 25 min (D), creating shallower gradients in the inter-wound region. (G–O) In vivo imaging (G–I) with representative hemocyte tracks; *srp-Gal4* drives *UAS-nuclear-red-stinger*. Two close wounds caused reduced bias in the inter-wound region (red and blue, J) while two wounds far apart behaved separately, with slightly reduced bias on the outer sides of the wing (L). For wounds at an intermediate distance, cell bias was significantly lower in the inter-wound region (K and N) for all time points examined (O), with clear hemocyte confusion in vivo (boxed cell tracks in H and plotted trajectories in M). Gray boxes indicate wound position (J–L). See also Figure S4 and Movies S4 and S5.

Knowing the spatial-temporal diffusion characteristics of the single-wound attractant (Figures 3C–3F), we can simulate in silico how chemoattractant gradients from two synchronously made wounds will interact (Figure 4; Movie S4). Here, our model also accounts for any boundary effects that may occur for wounds located close to the wing margin (see Experimental Procedures for details). For wounds that are close (centers only 150 μm apart; Figures 4A and 4B), our model predicts that the two attractant gradients will rapidly overlap (Figure 4A), and, after 25 min, the two gradients will mimic that of a single, very large wound (Figure 4B), resulting in less biased migration of hemocytes in the inter-wound region (Figures S4A and S4D). By contrast, for wounds that are far apart (480 μm), their attractant gradients will not interact with each other (Figures 4E and 4F). Our simulations predict that cells will respond to these individual distant wounds in almost the same manner as for two single wounds (Figures S4C and S4F), except that, because of pupal wing geometry, the two wounds both sit close to the outer margin of the wing, and our model predicts a stronger accumulation of attractant in the wing periphery compared to the inter-wound region, resulting in shallower gradients and less strongly biased cell movement on the outer sides.

Our simulations predict the exact intermediate distance that should cause maximal hemocyte “confusion” in the inter-wound region. At this distance apart (330 μm between centers; Figure 4C), the two attractant gradients would significantly overlap by 25 min (Figure 4D), causing much shallower gradients in the inter-wound region. In this scenario, the opposing gradients would be effectively balanced as the two wounds essentially compete for hemocyte attention; thus, our simulations predict a drop in hemocyte cell bias in this region (Figures S4B and S4E).

In order to test the validity of these in silico predictions, we generated large in vivo imaging datasets recording the inflammatory response to the different synchronous double-wound scenarios (Figures 4G–4I). As our model had predicted, two wounds made close together showed reduced bias in the inter-wound region during the first 20 min after wounding (red, Figure 4J), which further decreased over time (blue, Figure 4J; Figure S4G). Similarly, wounds generated far apart behaved as predicted (Figure 4L; Figure S4I), and we, indeed, observed a reduced bias on the wound sides closest to the margins, confirming that the wing geometry itself impacts the gradient shape.

Finally, for the wounds at an intermediate distance (330 μm apart), cell bias was significantly reduced in the inter-wound region as early as 20 min after wounding (Figure 4K, red; Figure S4H), indicating that opposing chemoattractant gradients had overlapped, as predicted, and caused significant hemocyte “confusion” in this region (see trajectory analysis in Figure 4M). Cells located between wounds had a significantly reduced bias compared to those in outer regions (Figure 4N) at all time points post-wounding (Figure 4O). Indeed, tracking of individual cell behaviors in vivo show cells turning back and forth (dithering) between the two wounds (Figure 4H, box; Figure 4M; Movie S5). Collectively, these experiments confirm the validity of the inferred attractant parameters and spatiotemporal gradient quantification.

### Modeling Repetitive Tissue Injury Reveals a Period of Hemocyte Desensitization

**Figure 5:**
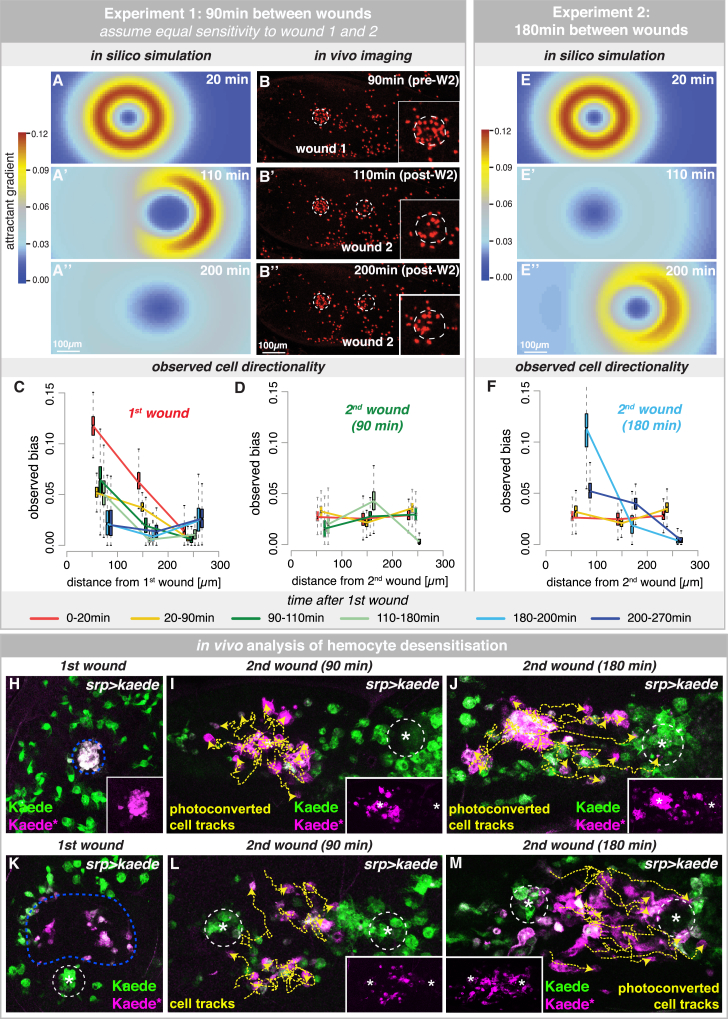
Modeling Repetitive Tissue Injury Uncovers a Period of Hemocyte Desensitization (A–D) Simulated interaction of attractants from wounds made 90 min apart, assuming that hemocytes respond to both wounds with equal sensitivity (A–A′′). Crescent shape of attractant gradient reflects impact of residual wound 1 attractant on newly made wound 2 (W2) (A′). In vivo time-lapse imaging (B–B′′; *srp>nuclear-red-stinger*) confirms a normal response to the first wound (C) but reveals a significantly reduced response to the second wound, more similar to unwounded tissues (D). (E and F) Simulated interaction of attractant gradients for two wounds made 3 hr apart, assuming hemocytes are fully resensitized to the attractant (E–E′′). In vivo imaging confirms this prediction (F). In (C, D, and F), boxplots represent the marginal posterior parameter distributions for the observed bias estimated from extracted cell trajectories for each spatiotemporal cluster. (H–M) *srp-Gal4* drives expression of photoconvertible fluorophore Kaede (green) in hemocytes. Kaede photoconversion (majenta) tags hemocytes localized at (H–J) or adjacent to (K–M) the first wound. Tagged hemocytes (magenta; also see insets) are blind to a second wound made 90 min after the initial injury (I and L) but drawn to a second wound made 3 hr later (J and M). Representative tracks (yellow) of tagged cells show hemocyte behavior following the second wound. See also Figure S5.

Our in silico analysis of multiple chemoattractant sources presented an ideal opportunity to investigate how cells behave in response to repetitive tissue damage. Here, we tested whether hemocyte exposure to a first wave of pro-inflammatory mediator, released from an initial wound, would change hemocyte behavior in response to a later second wound (Figure 5). Using the attractant parameters extracted from single wounds, we simulated how the attractant gradients from a first wound and from a second wound made 90 min later would overlap in space and time (Figures 5A–5A′′). In the absence of a priori knowledge, our model assumes that hemocytes will respond with equal sensitivity to first and second wounds. Given that individual wounds release new wound signal for approximately only 30 min post-injury, the first wound is predicted to have only a minor impact on the response to the newly made second wound (Figures 5A–5A′′).

To test these predictions in vivo, we generated large datasets of in vivo time-lapse imaging of two sequential wounds made 90 min apart (Figures 5B–5B′′). Hemocyte trajectories were analyzed, and directional bias was computed for each spatiotemporal cluster as before (Figures 5C and 5D). As expected, hemocytes responded rapidly to the first wound with the same spatiotemporal dynamics as for single wounds (Figure 5C). However, the hemocyte response to the second wound is significantly attenuated, and hemocytes exhibited only low levels of cell bias, similar to that of unwounded samples (Figure 5D). This suggests that exposure to the first wound signal desensitized hemocytes to subsequent tissue injury, and they essentially ignored the presence of the second wound. To test whether hemocyte desensitization is temporary, we generated new in vivo imaging datasets in which the second wound was created 3 hr after the initial wound. Here, the hemocyte spatiotemporal cell bias in response to the second wound was restored to baseline levels, as predicted if hemocytes had regained full wound sensitivity (Figures 5E–5F).

We further explored this phenomenon in vivo by selectively labeling hemocytes that had responded to the first wound using the photoconvertible fluorophore Kaede [30], labeling cells that had already reached the wound site (Figure 5H; Figure S5) or those nearby (Figure 5K). We found that the photoconverted cells do not turn and migrate toward a second wound made 90 min after the initial injury (Figures 5I and 5L), but they are successfully drawn to a second wound 3 hr later (Figures 5J and 5M). These data suggest that the hemocyte wound refractory period is short lived, and cells become resensitized to new wound attractant within 3 hr of the initial injury.

### Modeling Chronic Inflammation Uncovers Dramatic Hemocyte Behavioral Changes Associated with Non-healing Wounds

In all wounding conditions analyzed so far, hemocytes are highly biased toward the injury site—even those experiencing the highest attractant levels adjacent to the wound edge—suggesting hemocytes have not undergone receptor saturation. However, we hypothesize that, as we consider even larger wounds, the total attractant concentration will scale with wound diameter, and we could theoretically reach a wound size that generates sufficiently high attractant levels to cause receptor saturation. Here, receptor-saturated hemocytes close to the wound might lose their orientation within the attractant field and lack directional bias toward the wound.

**Figure 6:**
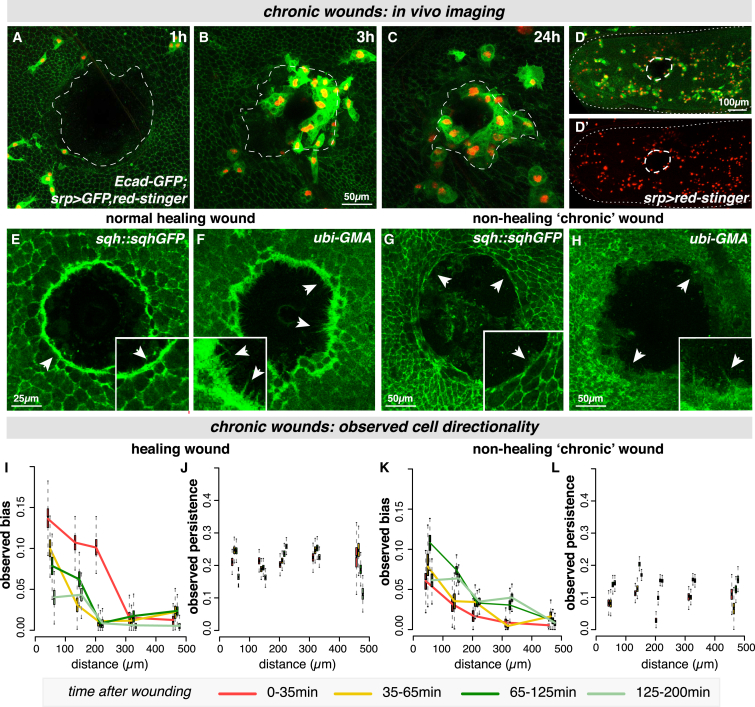
Distinct Hemocyte Behaviors Associated with Non-healing Wounds (A–D′) In vivo imaging of extra-large “chronic” wounds (130-μm diameter) that fail to heal and remain open 24 hr post-injury (A–C; wound edge outlined in white) with low-level persistent inflammation (B–D). Epithelium labeled with *E-cadherin-GFP* and hemocytes with *srp > nuclear-red-stinger, GFP*. Data from live-imaging in (D′) used to compute hemocyte directionality. (E–H) Normal healing wounds close using a contractile acto-myosin cable (*sqh-GFP*, arrowheads, E) and leading-edge protrusions (*GFP-moesin,* arrowheads, F), but chronic “non-healing” wounds lack a stable actin cable (arrowheads, G) and have only rare protrusions (arrowheads, H). (I–L) For healing wounds, hemocytes respond with similar levels of bias and persistence as for previous large wounds (I and J). Hemocytes associated with “non-healers” exhibited little or no bias toward the wound (K), even at the earliest time points (red line, K), and significantly less persistence (L). Boxplots represent estimated parameter distributions for bias and persistence. See also Figure S6 and Movie S6.

To test whether this phenomenon can be observed in vivo, we developed a wounding strategy to generate extra-large (130 μm diameter) wounds (Figure 6). Hemocyte trajectories were extracted from our imaging data and used to compute cell bias and persistence (Figures 6I–6L). Surprisingly, for extra-large wounds that healed, hemocytes behaved in a manner similar to that observed previously for other single wounds, with the strongest bias appearing in the first 30 min after wounding close to the wound (Figure 6I) and no change in cell persistence (Figure 6J). The attractant concentrations reached in these extra-large wounds in vivo are, therefore, insufficient to cause detectable hemocyte receptor saturation in vivo.

However, for these extra-large wounds, we observe a striking and unexpected level of heterogeneity in their healing ability (Figures 6A–6H). Although, occasionally, these wounds healed after some delay, the majority of the extra-large wounds fail to undergo the normal repair process, and the epithelial hole remains open even 24 hr post-injury (Figures 6A–6C; Movie S6). To investigate the cellular mechanism underpinning this dramatic non-healing phenotype, we analyzed the actin repair machinery in these non-healing wounds (Figures 6E–6H; Figure S6). Unlike healing wounds that close using a combination of a leading-edge actomyosin cable and dynamic actin-rich protrusions in the front-row cells (Figures 6E and 6F; Figures S6F–S6J) [31], we find that, although non-healing wounds initially assemble an actin cable (and, at first, appear indistinguishable from their healing counterparts), the cable is not maintained (Figure 6G; Figures S6A–S6E), filopodia are rarely extended from the leading edge (Figure 6H), and the wounds fail to close.

These non-healing wounds also exhibit a low-level ongoing inflammation for the full period of imaging (Figures 6B–6D). These phenomena—failure in repair and non-resolving inflammation—are hallmarks of chronic non-healing wounds [1, 14]. To our knowledge, this is the first example of chronic wounds in a genetically tractable model and provides a unique opportunity to study this poorly understood clinical condition. Such wound heterogeneity is a key factor in clinical scenarios of chronic wounds [14], and a major goal of wound healing research is to uncover prognostic indicators that might predict whether a wound is likely to heal or be a stubborn “non-healer” [1, 14]. Here, we can exploit the heterogeneity of our chronic wounds and the dynamic imaging of the associated hemocyte behavior to potentially offer some insight into this phenomenon.

Hemocytes associated with the subsequently non-healing (chronic) wounds behaved in a strikingly different manner from that observed in similar-sized wounds that healed successfully (compare Figures 6I and 6J with 6K and 6L). From the earliest time points in non-healing wounds, hemocytes failed to exhibit a strong bias toward the wound; this was particularly evident in the first 30 min post-injury (Figure 6K, red line). Furthermore, hemocytes in the vicinity of the chronic wound migrated with significantly less persistence (Figure 6L), whereas healing wounds exhibited no apparent change from baseline levels of cell persistence from earliest stages (Figures 2 and 6J). Our data suggest prognostic criteria in the early behavior of hemocytes, which allow us to predict in the first hour post-wounding whether a wound is likely to successfully heal or become chronic.

## Discussion

**Figure 7:**
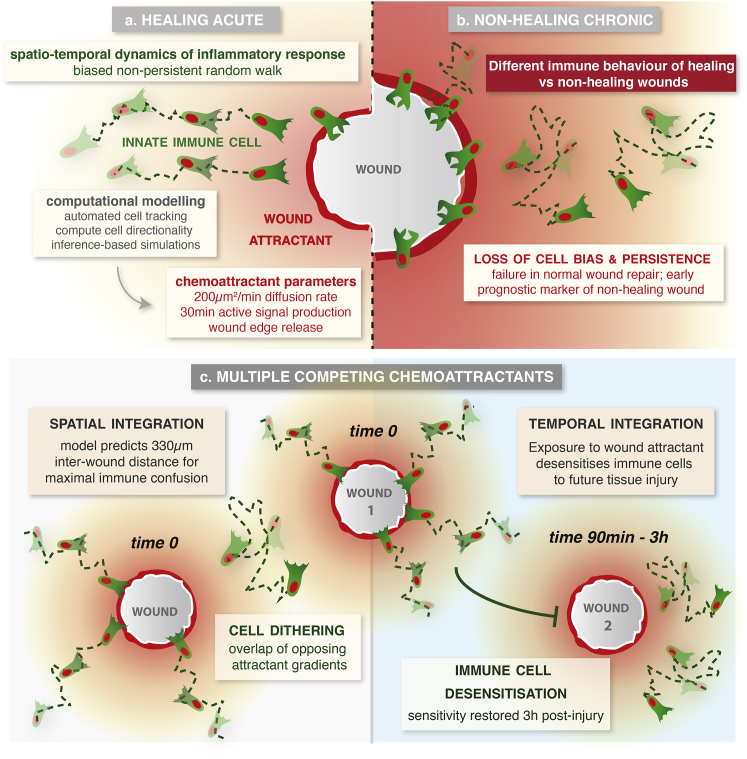
Modeling the In Vivo Inflammatory Response to Single Acute, Chronic, and Competing Wounds Computational modeling uncovered the spatiotemporal dependence of immune cell behavior in response to wounding in vivo, revealing a wave-like cell response that enabled quantification of the wound attractant gradient (a). For extra-large wounds that fail to heal (b), immune cells behave dramatically differently, exhibiting very low bias and persistence even from earliest stages. Using these parameters, we model more complex immune behavior (c), predicting the inter-wound distance to generate maximal immune cell confusion due to spatial integration of overlapping attractants and revealing a temporary desensitization period after initial wound exposure.

Although recent advances in microscopy techniques have allowed us to visualize the acute inflammatory response to tissue damage with high spatiotemporal resolution [2, 3], and genetic approaches in model organisms have identified putative immune attractants [5, 6, 7], it remains impossible to directly observe and quantify these chemoattractant gradients in vivo. In this study, we developed a novel integrative approach, using statistical modeling to extract detailed information about the wound attractant signals from our in vivo imaging data of immune cell behavior. Using our simulation models, we could translate the experimentally observed immune cell trajectories to infer previously unknown and otherwise experimentally inaccessible details of the pro-inflammatory wound attractant, and we could use these new signaling parameters to model immune cell behavior in more complex wound scenarios, e.g., fields of competing attractants (Figure 7).

In particular, we show that the wound attractant signal is actively released from the wound edge for 30 min post-wounding, independent of wound size, and diffuses across the tissue at a rate of 200 μm^2^/min. A comparison of these inferred attractant parameters with those of previously implicated damage signals, such as ATP and H_2_O_2_, suggests that these cannot be the primary attractants responsible for hemocyte wound recruitment in vivo; indeed, the published diffusion coefficients of ATP and H_2_O_2_ are 18,000 μm^2^/min (ATP in water), 9,000 μm^2^/min (ATP in cytoplasm), and 84,000 μm^2^/min (H_2_O_2_ in water) [28, 29, 32, 33], indicating that both diffuse at rates significantly faster than that of the wound attractant identified in our model. This suggests that ATP and H_2_O_2_ may, therefore, establish a pro-inflammatory permissive environment to potentiate the recruitment of immune cells into the inflamed tissue, with additional chemotactic signals required to direct cells into the precise area of damage. For example, there is some evidence to suggest that ATP enhances the immune cell response to a pre-existing attractant by generating a signal amplification loop [34] or by promoting neutrophil adhesion within the vasculature [4], although our own RNAi knockdown experiments of the only *Drosophila* ATP receptor, Ado-R, suggest no role for ATP as a pupal wound attractant for hemocytes. However, recent genetic evidence from *Drosophila* also suggests that H_2_O_2_ potentiates the immune cell response to injury by activating the damage receptor Draper [11] rather than operating as a direct attractant.

Our new insight into the spatiotemporal properties of the in vivo wound attractant provides a starting point to identify credible candidates for the attractant signal. Flies are not known to have platelet-like cells (which could play a role in the release of damage signals in vertebrates), suggesting that the wound attractant in our system is a growth factor or DAMP released by damaged cells at the wound margin. A useful comparison is that of our inferred attractant parameters with those of other known developmental morphogens—particularly those that diffuse within similar tissue environments—to form predictions about the likely molecular weight and propagation mechanisms. The 50-kDa protein FGF8 (fibroblast growth factor 8)-EGFP, for example, moves by free Brownian diffusion in the extracellular space at a rate of 3,180 μm^2^/min in zebrafish embryos [35], 16× faster than our wound attractant; the transforming growth factor (TGF)-β ligand Dpp spreads much more slowly, at 6 μm^2^/min, in *Drosophila* wing discs (33× slower than our wound attractant), despite being of similar molecular weight to FGF8, as this spreads by transcytosis through target cells [36]. Alternative candidates for the wound attractant include the nuclear protein HMGB1, the heat shock family proteins (Hsps), mitochondrial-derived N-formyl peptides, and mitochondrial DNA, all of which have been implicated in the inflammatory immune response [6, 37].

During the vertebrate inflammatory response, there are presumed to be multiple overlapping signals that orchestrate the recruitment of leukocytes into the wound site [4, 12, 38]. While an intravascular gradient of chemokines guides leukocytes toward the vicinity of tissue damage, additional dominant “end-target” attractants recruit cells to the precise site of injury [4]. Here, we used our computational approach to model such complex multistep immune cell behavior in response to overlapping attractant signals. Using the spatiotemporal properties of the wound attractant learned from our single-wound analysis, we could accurately simulate how attractant gradients from two adjacent wounds would interact in space and time and predict the inter-wound distance that would cause maximal hemocyte disorientation (dithering behavior) in the inter-wound region. This experiment served as an important in vivo validation of our model and enabled us to verify the attractant gradient parameters that had been inferred from the hemocyte response to single wounds.

As immune cells in vivo encounter competing attractants not just in space but also in time, we also modeled the interaction of attractant gradients from two sequential wounds. We find that exposure to even low levels of the first wound attractant in vivo temporarily desensitizes hemocytes to detection of further tissue damage, although sensitivity is restored by 3 hr after the initial injury. Such ligand-dependent receptor desensitization, in which ligand-bound receptors are “switched off” after a transient period of signaling, is a common feature of G-protein-coupled chemokine receptors [39, 40]. Given that leukocytes must often migrate through fields of competing attractants, requiring them to prioritize new “end-stage” attractants over other “intermediate” ones [9, 12, 13], this desensitization phenomenon could be an important mechanism to ensure that leukocytes are able to move away from one chemoattractant field into another for effective chemotaxis. Indeed, Lin and Butcher [41] predicted, using an in silico approach, that homologous receptor desensitization would be essential for efficient navigation in fields of competing attractants; in the absence of desensitization, cells would remain oriented toward local signals and fail to respond to cues from more distant sources.

In our studies of extra-large wounds, we have also uncovered a new model for studying the chronic inflammation associated with non-healing wounds, a poorly understood phenomenon of major clinical importance [1, 14]. Just as in the clinic, we find heterogeneity in the healing ability of extra-large wounds in vivo in our experimental *Drosophila* model. Strikingly, we find a dramatic difference in the immune cell behavior associated with these different healing abilities, even at early stages before the healing status of the wound is clear. Hemocytes associated with large wounds that will eventually heal behave in a similar manner to that observed for smaller wounds, being strongly biased toward the wound, particularly in the first 30 min post-wounding. However, hemocytes associated with wounds that become chronic and fail to heal exhibit very little bias toward the injury site, even at early stages, and also migrate with significantly less persistence than their healing counterparts. Given that current diagnostic markers are largely based on the analysis of fixed tissue biopsy patient samples [14], our data now suggest that live imaging of the dynamic inflammatory response in patient wounds, perhaps after fluorescence-activated cell sorting (FACS) of immune cells, could provide a valuable prognostic indicator for predicting whether an injury will likely heal or become chronic.

This study highlights the valuable insights that can be extracted from in vivo imaging data if computational analysis approaches are used in situations that would otherwise remain experimentally inaccessible. While we have focused on the pro-inflammatory mediators orchestrating the immune response to tissue damage, this type of statistical methodology could be applied to other biological questions that are challenging for direct measurement or observation [42, 43, 44, 45]; one important application is the design of optimally informative experiments to study complex biological processes, which is particularly important when conducting experiments in animals. We envision that such integrative approaches will continue to advance our understanding of biological phenomena at rates unattainable by experimental biologists alone.

## Experimental Procedures

### *Drosophila* Stocks and Genetics

Fly stocks were maintained according to standard protocols [46]. The following lines were used: *E-cadherin-GFP* [47], *UAS-GFP*, *UAS-rpr*, *UAS-GFP-Moesin*, *UAS-adoR-RNAi*, *UAS-mCherry-Moesin*, *sqh-GFP*, and *sqh*^*AX3*^ (Bloomington Drosophila Stock Center); *UAS-kaede* (a gift from Wes Grueber [30]); *srp-Gal4* (a gift from Katja Bruckner, University of California, San Francisco [48]); and *UAS-nuclear-red-stinger* (a gift from Brian Stramer, King’s College London [26]).

### Microscopy and Wounding

Pupae were aged to the appropriate developmental stage (16 hr APF, unless otherwise stated) in a glass vial at 25°C. Pupae were carefully removed from their protective casing with forceps and microscissors before being mounted on a glass coverslip using heptane glue. Wounds were induced using a nitrogen-pumped Micropoint ablation laser tuned to 435 nm (Andor Technologies) [10]. Bead micro-injection was performed as described previously [49]. Imaging was performed on a Leica TCS SP5 confocal microscope. Photoconversion was performed using the Leica FRAP software and a 405-nm laser [50].

### Statistical Data Analysis

Cells were automatically extracted and tracked from all images, and resulting cell trajectories were clustered and analyzed. Bias and persistence of cells, as well as the attractant gradient, were determined using Bayesian inference [21, 22] to estimate the parameters of a bias-persistent random walk model and of a 2D diffusion model. All displayed simulations are the mean of 500 simulations, with parameters drawn from the inferred posterior parameter distributions. Detailed information about all data-processing steps, models, and inference procedures are given in the Supplemental Information.

## Author Contributions

H.W. performed the in vivo experiments. J.L. developed the in silico methods and performed the computational analysis. H.W. and J.L. contributed to the design of the study and to the writing of the manuscript. A.S. contributed to the development of in silico methods and proofread the manuscript. W.W. helped write the manuscript. P.M. and M.P.H.S. were the principal investigators who designed the study, coordinated the project, and helped write the manuscript.

## Supplemental Information

Document S1. Figures S1–S6 and Supplemental Experimental Procedures

Movie S1. The Acute Inflammatory Response to Tissue Wounding in the *Drosophila* Pupal Wing, Related to Figure 1 Time-lapse movie of the dynamic behavior of *Drosophila* immune cells (hemocytes) in response to tissue wounding. Epithelial cells are labeled using E-cadherin-GFP (green cell outlines), immune cell nuclei are labeled using nuclear Red-Stinger (red) and immune cell cytoplasm using cytoplasmic GFP (green) both driven by *srp-Gal4*. Upon laser-induced wounding to the pupal wing epithelium, immune cells are rapidly recruited towards the damage site during the first 2 hours post-wounding in a strict spatiotemporal manner and accumulate within the wounded area, where they phagocytose necrotic cell debris. Scale bar represents 20μm.

Movie S2. Conditions Used to Analyze Spatiotemporal Dynamics of Immune Cell Behavior before and after Wounding, Related to Figure 1 In vivo time-lapse movies of the dynamic behavior of *Drosophila* immune cells in control unwounded tissue (left) and in response to tissue wounding (center and right, for small and large wounds, respectively) generated for later 3D tracking and computational analysis. Epithelial cells are labeled using E-cadherin-GFP (green cell outlines, lower panels) whilst immune cell nuclei are labeled using nuclear Red-Stinger (red, upper and lower panels) and cytoplasmic GFP (green, lower panels) driven by *srp-Gal4*. In the absence of wounding, immune cells migrate randomly beneath the wing epithelium (left). Upon laser-induced wounding (asterisks), immune cells are rapidly recruited towards the damage site in a strict spatiotemporal manner (center and right); the size of the response scales with wound size, with more robust recruitment to larger wounds (right). Scale bar represents 75μm for upper and 100 μm for lower panel images.

Movie S3. Inferred Spatiotemporal Dynamics of the Immune Attractant Gradient Generated upon Tissue Damage, Related to Figure 3 The inferred spatiotemporal diffusion gradient of the wound attractant, according to the model in which the attractant emanates from the wound edge, for small (upper panel) and large (lower panel) wounds. Units on x- and y-axes are in μm. Colours (shown at the scale bar) represent attractant concentrations relative to the highest predicted concentration, i.e. ranging from 0% to 100%.

Movie S4. Simulated Spatiotemporal Interaction of Wound Attractant Gradients from Two Competing Wounds at Different Inter-wound Distances, Related to Figure 4 The predicted spatiotemporal interaction of wound attractant diffusion gradients from two large adjacent wounds, according to the model in which the attractant emanates from the wound edge. Wounds are positioned close together (150μm distance between wound centers, top), at intermediate distance (330μm distance between wound centers, middle) and far apart (480μm distance between wound centers, bottom). Units on the x- and y-axes are in μm. Colours (shown at the scale bar) represent attractant concentrations relative to the highest predicted concentration, i.e. ranging from 0% to 100%. Simulations take into account boundary effects resulting from the wing geometry.

Movie S5. Spatial Integration of Attractant Gradients from Two Competing Wounds Causes Immune Cell “Dithering” Behavior, Related to Figure 4 In vivo time-lapse imaging of the dynamic behavior of *Drosophila* hemocytes in response to two competing wounds (asterisks) generated simultaneously in the wing epithelium (labeled with *Ecadherin-GFP*). At this inter-wound distance (330μm), opposing attractant gradients rapidly overlap in the inter-wound region and this causes significantly reduced hemocyte bias towards either wound. Hemocytes situated in this region often become ‘confused’ and ‘dither’, moving back and forth, without strong attraction towards either wound (see representative multicolour hemocyte tracks). Here, hemocyte nuclei are labeled using nuclear Red-Stinger (red) driven by *srp-Gal4*. Scale bar represents 100μm.

Movie S6. In Vivo Model of the Inflammatory Response to Chronic Non-healing Wounds, Related to Figure 6 in vivo imaging of extra-large wounds that fail to heal and remain open even 24 hours post-injury. Unlike normal healing wounds, the inflammatory response to these non-healing wounds is significantly attenuated, even at the earliest stages post-wounding, as hemocytes migrate with significantly less persistence and bias towards the wound site than is normally observed in the acute inflammatory response (compare with Movie S1). Epithelial cells are labeled using E-cadherin-GFP (green cell outlines), immune cell nuclei are labeled using nuclear Red-Stinger (red) and immune cell cytoplasm using cytoplasmic GFP (green) both driven by *srp-Gal4*. Scale bar represents 20μm.

Document S2. Article plus Supplemental Information
