# Supplementary figures and images for: Systems Analysis of the Dynamic Inflammatory Response to Tissue Damage Reveals Spatiotemporal Properties of the Wound Attractant Gradient
Source: Curr Biol. 2016 Aug 8;26(15):1975–89. doi: 10.1016/j.cub.2016.06.012 (PMC4985561; doi:10.1016/j.cub.2016.06.012)

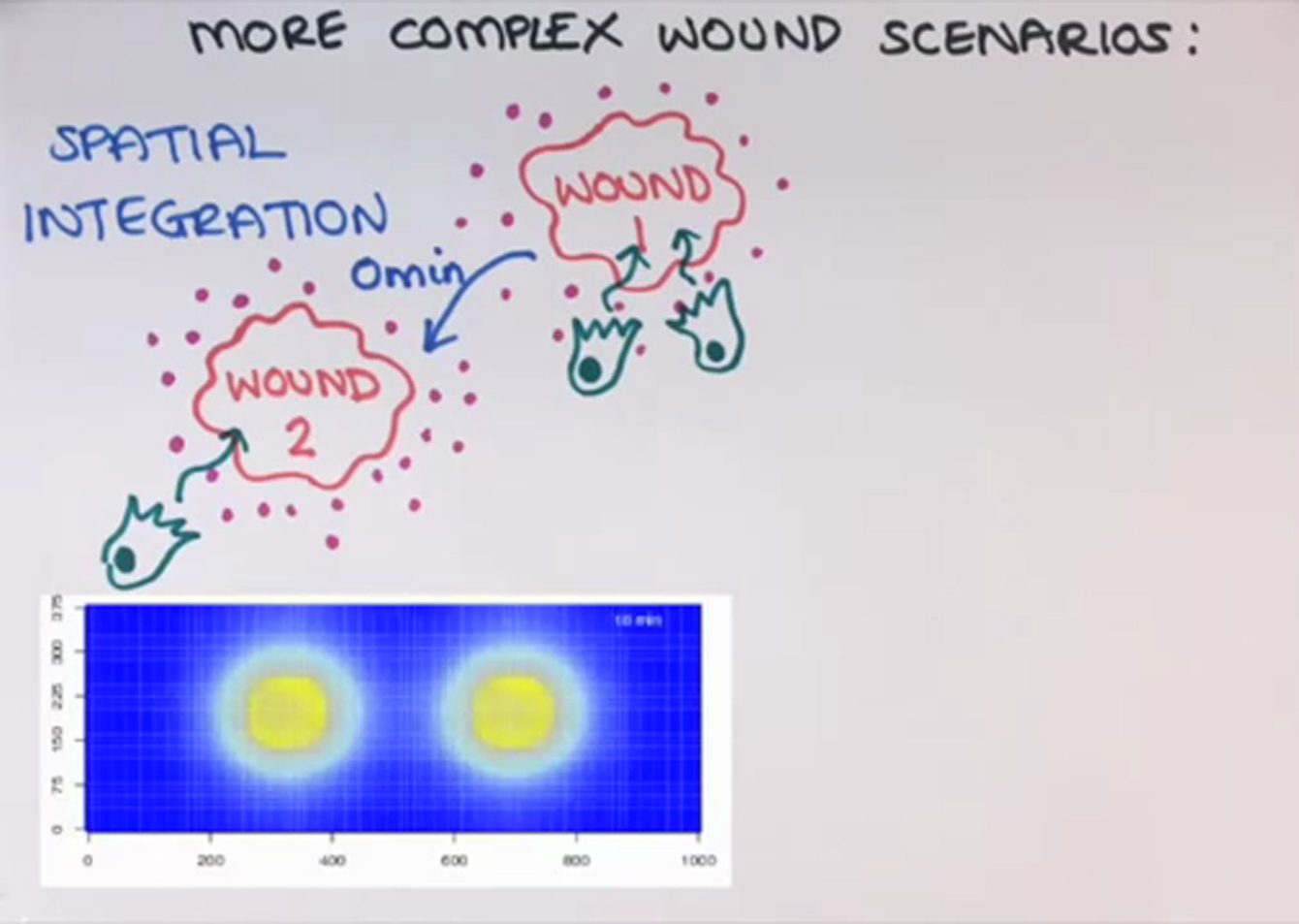

Supplement: Supplementary file 1 [file mmc9.jpg]

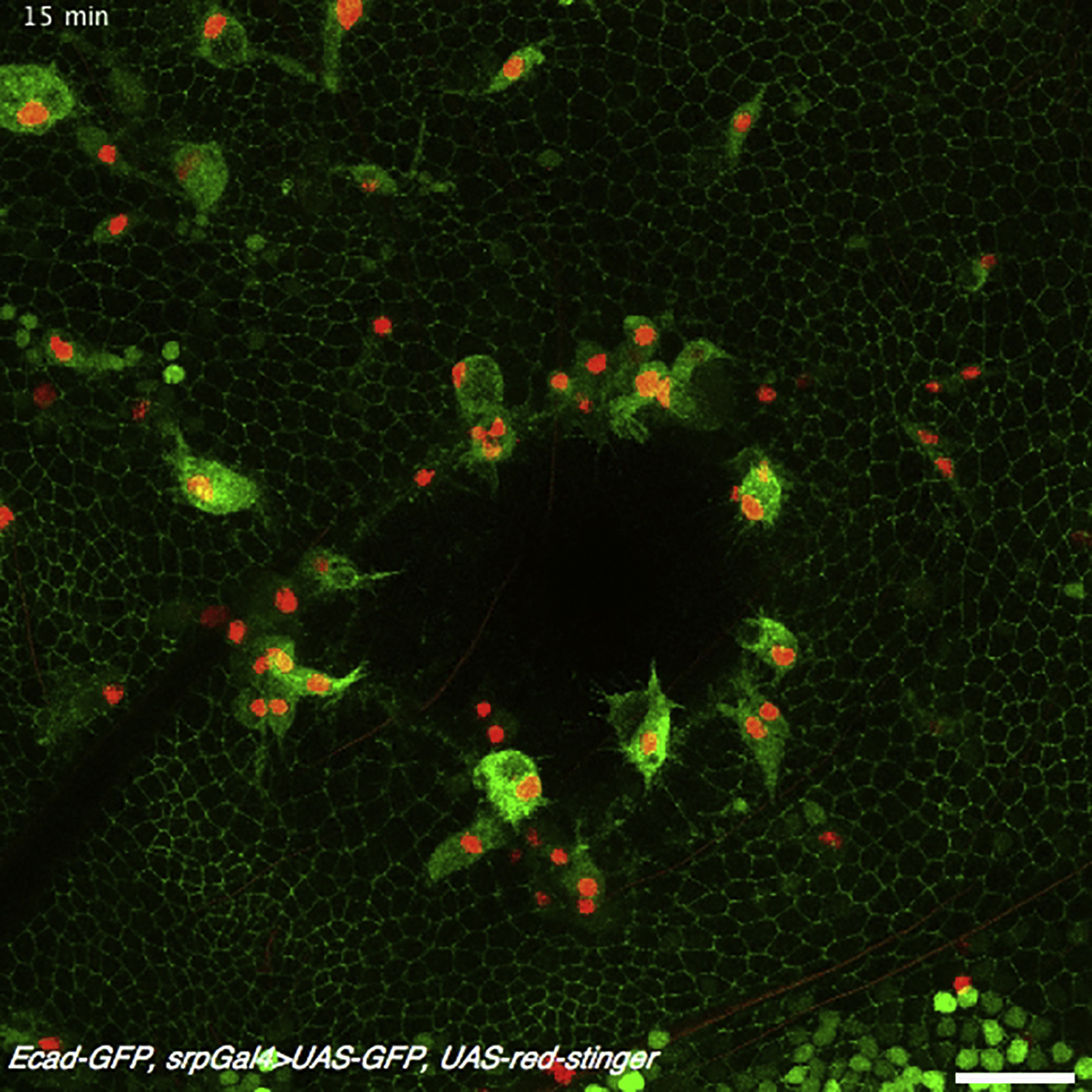

Supplement: Movie S1. The Acute Inflammatory Response to Tissue Wounding in the Drosophila Pupal Wing, Related to Figure 1 — Time-lapse movie of the dynamic behavior of Drosophila immune cells (hemocytes) in response to tissue wounding. Epithelial cells are labeled using E-cadherin-GFP (green cell outlines), immune cell nuclei are labeled using nuclear Red-Stinger (red) and immune cell cytoplasm using cytoplasmic GFP (green) both driven by srp-Gal4. Upon laser-induced wounding to the pupal wing epithelium, immune cells are rapidly recruited towards the damage site during the first 2 hours post-wounding in a strict spatiotemporal manner and accumulate within the wounded area, where they phagocytose necrotic cell debris. Scale bar represents 20μm. [file mmc2.jpg]

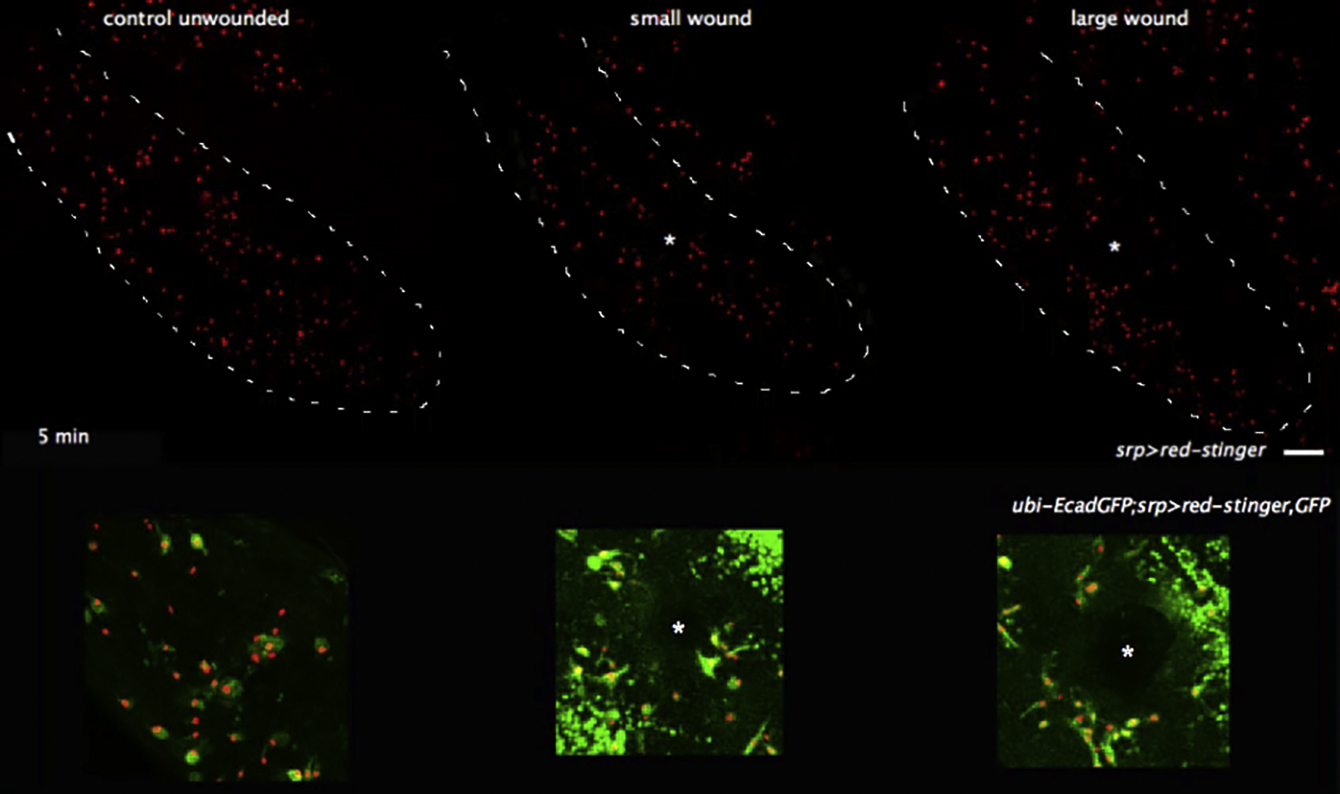

Supplement: Movie S2. Conditions Used to Analyze Spatiotemporal Dynamics of Immune Cell Behavior before and after Wounding, Related to Figure 1 — In vivo time-lapse movies of the dynamic behavior of Drosophila immune cells in control unwounded tissue (left) and in response to tissue wounding (center and right, for small and large wounds, respectively) generated for later 3D tracking and computational analysis. Epithelial cells are labeled using E-cadherin-GFP (green cell outlines, lower panels) whilst immune cell nuclei are labeled using nuclear Red-Stinger (red, upper and lower panels) and cytoplasmic GFP (green, lower panels) driven by srp-Gal4. In the absence of wounding, immune cells migrate randomly beneath the wing epithelium (left). Upon laser-induced wounding (asterisks), immune cells are rapidly recruited towards the damage site in a strict spatiotemporal manner (center and right); the size of the response scales with wound size, with more robust recruitment to larger wounds (right). Scale bar represents 75μm for upper and 100 μm for lower panel images. [file mmc3.jpg]

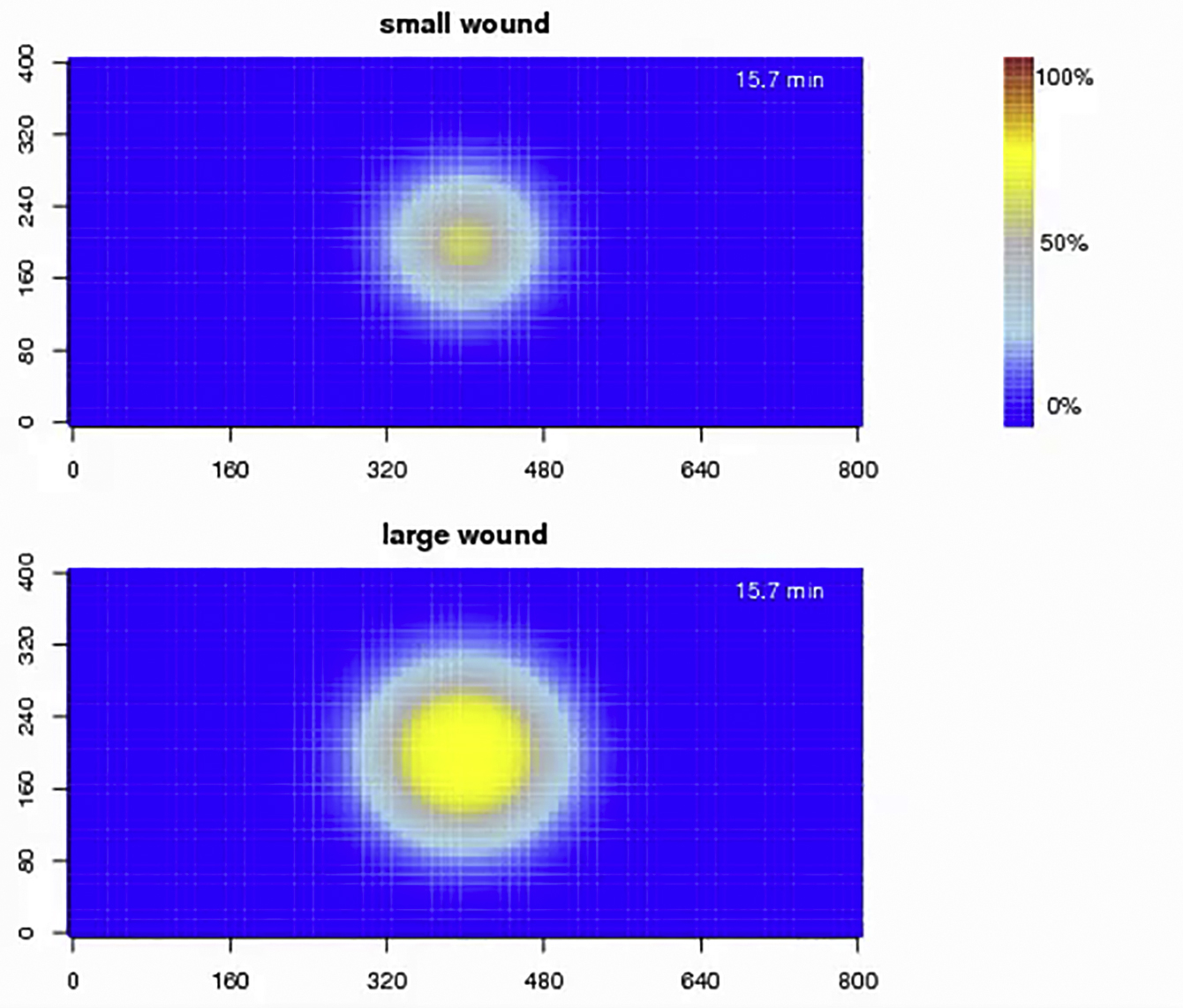

Supplement: Movie S3. Inferred Spatiotemporal Dynamics of the Immune Attractant Gradient Generated upon Tissue Damage, Related to Figure 3 — The inferred spatiotemporal diffusion gradient of the wound attractant, according to the model in which the attractant emanates from the wound edge, for small (upper panel) and large (lower panel) wounds. Units on x- and y-axes are in μm. Colours (shown at the scale bar) represent attractant concentrations relative to the highest predicted concentration, i.e. ranging from 0% to 100%. [file mmc4.jpg]

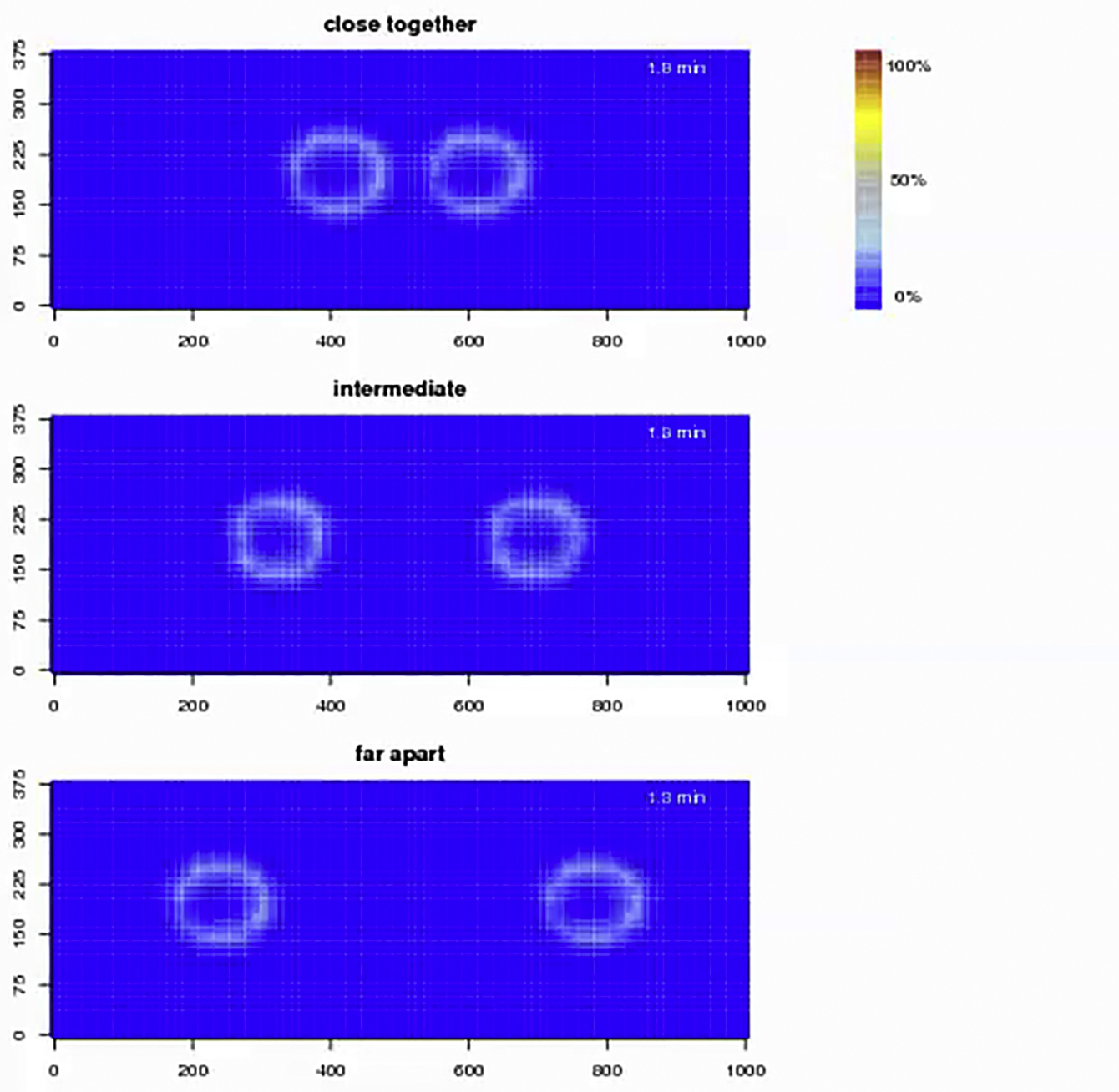

Supplement: Movie S4. Simulated Spatiotemporal Interaction of Wound Attractant Gradients from Two Competing Wounds at Different Inter-wound Distances, Related to Figure 4 — The predicted spatiotemporal interaction of wound attractant diffusion gradients from two large adjacent wounds, according to the model in which the attractant emanates from the wound edge. Wounds are positioned close together (150μm distance between wound centers, top), at intermediate distance (330μm distance between wound centers, middle) and far apart (480μm distance between wound centers, bottom). Units on the x- and y-axes are in μm. Colours (shown at the scale bar) represent attractant concentrations relative to the highest predicted concentration, i.e. ranging from 0% to 100%. Simulations take into account boundary effects resulting from the wing geometry. [file mmc5.jpg]

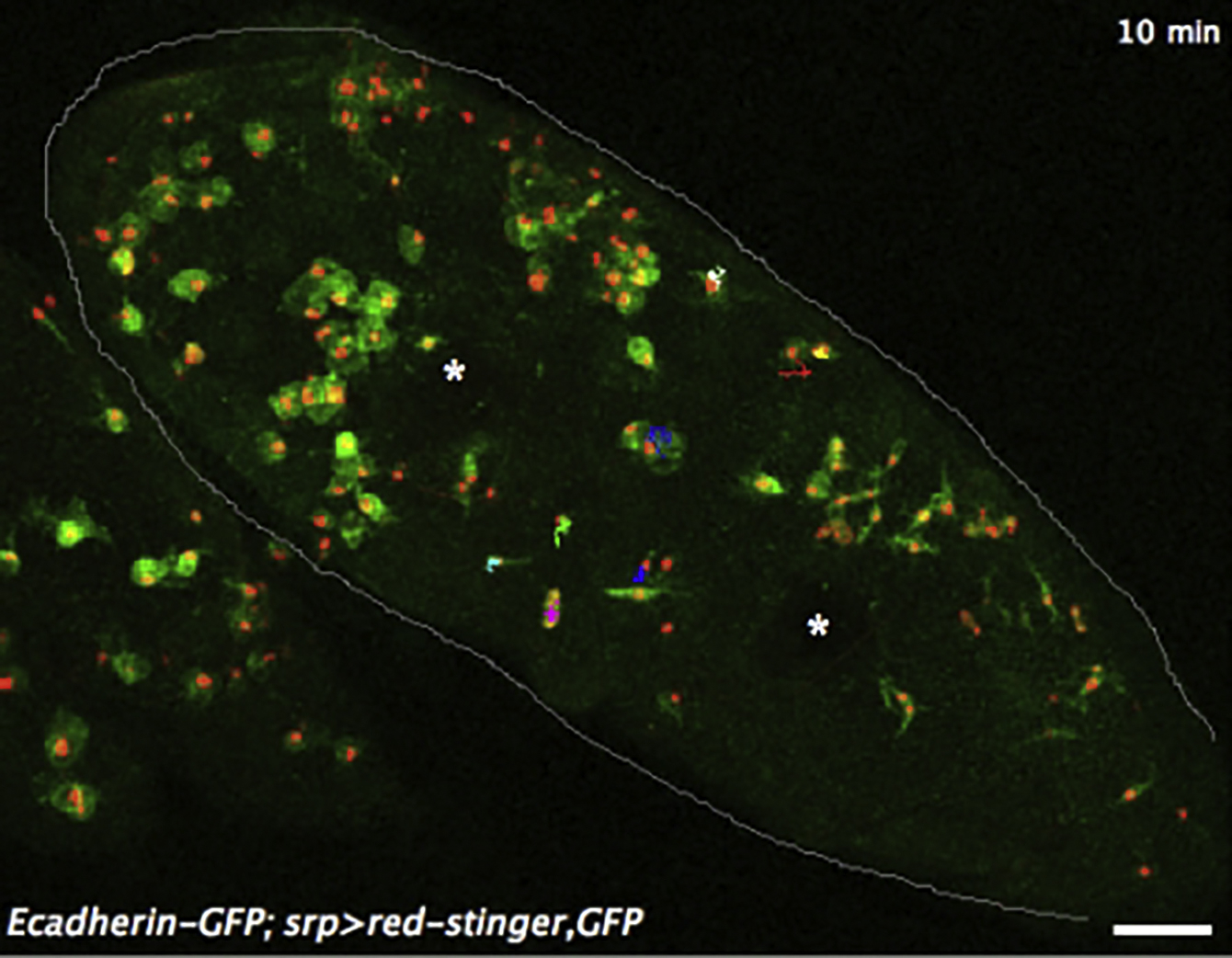

Supplement: Movie S5. Spatial Integration of Attractant Gradients from Two Competing Wounds Causes Immune Cell “Dithering” Behavior, Related to Figure 4 — In vivo time-lapse imaging of the dynamic behavior of Drosophila hemocytes in response to two competing wounds (asterisks) generated simultaneously in the wing epithelium (labeled with Ecadherin-GFP). At this inter-wound distance (330μm), opposing attractant gradients rapidly overlap in the inter-wound region and this causes significantly reduced hemocyte bias towards either wound. Hemocytes situated in this region often become ‘confused’ and ‘dither’, moving back and forth, without strong attraction towards either wound (see representative multicolour hemocyte tracks). Here, hemocyte nuclei are labeled using nuclear Red-Stinger (red) driven by srp-Gal4. Scale bar represents 100μm. [file mmc6.jpg]

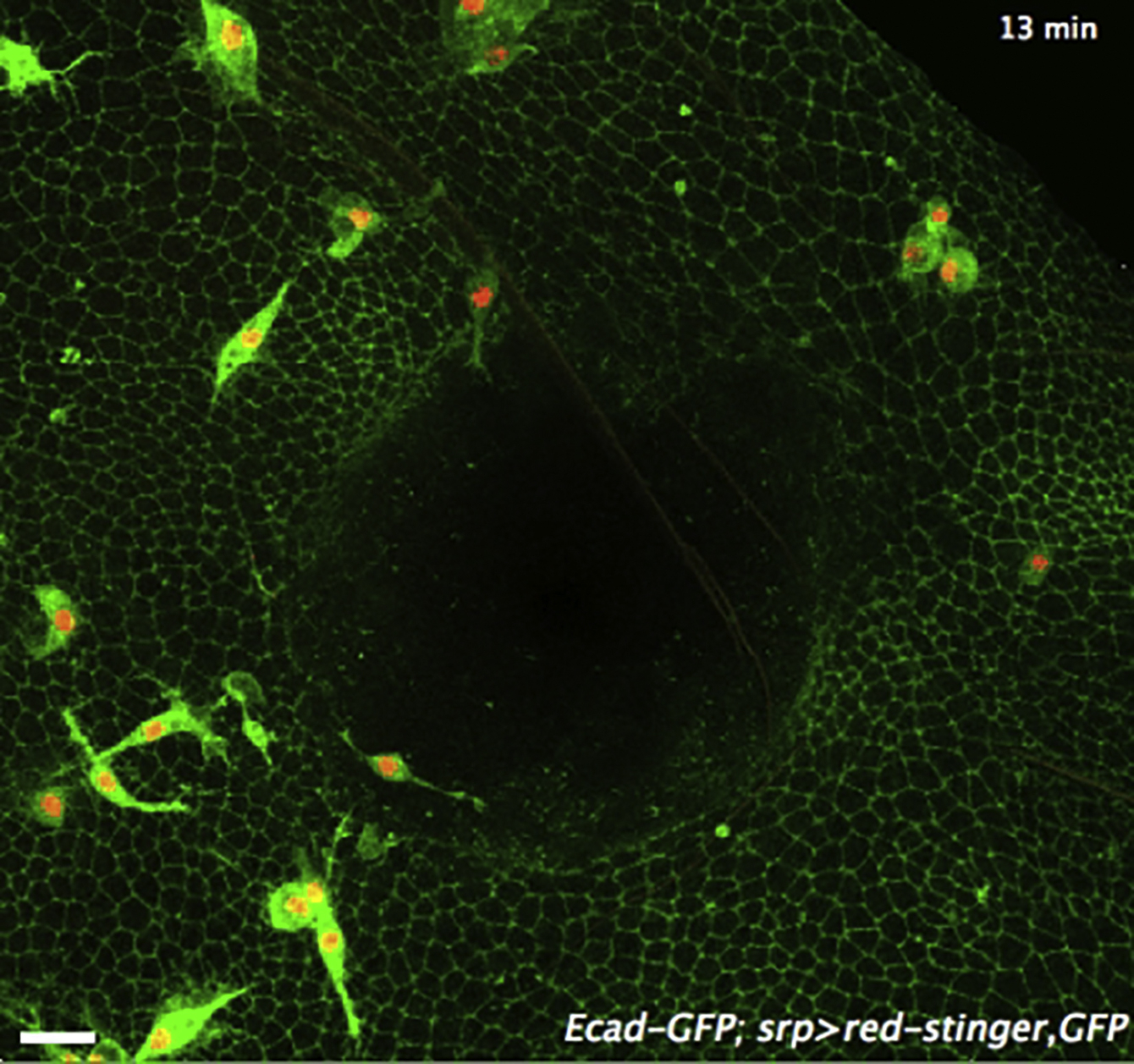

Supplement: Movie S6. In Vivo Model of the Inflammatory Response to Chronic Non-healing Wounds, Related to Figure 6 — in vivo imaging of extra-large wounds that fail to heal and remain open even 24 hours post-injury. Unlike normal healing wounds, the inflammatory response to these non-healing wounds is significantly attenuated, even at the earliest stages post-wounding, as hemocytes migrate with significantly less persistence and bias towards the wound site than is normally observed in the acute inflammatory response (compare with Movie S1). Epithelial cells are labeled using E-cadherin-GFP (green cell outlines), immune cell nuclei are labeled using nuclear Red-Stinger (red) and immune cell cytoplasm using cytoplasmic GFP (green) both driven by srp-Gal4. Scale bar represents 20μm. [file mmc7.jpg]
