# Supplementary material for: Systems Analysis of the Dynamic Inflammatory Response to Tissue Damage Reveals Spatiotemporal Properties of the Wound Attractant Gradient
Source: Curr Biol. 2016 Aug 8;26(15):1975–89. doi: 10.1016/j.cub.2016.06.012 (PMC4985561; doi:10.1016/j.cub.2016.06.012)
Supplement: Document S1. Figures S1–S6 and Supplemental Experimental Procedures [file mmc1.pdf]

Current Biology, Volume *26*

Supplemental Information

Systems Analysis of the Dynamic Inflammatory Response to Tissue Damage Reveals Spatiotemporal Properties of the Wound Attractant Gradient

Helen Weavers, Juliane Liepe, Aaron Sim, Will Wood, Paul Martin, and Michael P.H. Stumpf

Figure S1

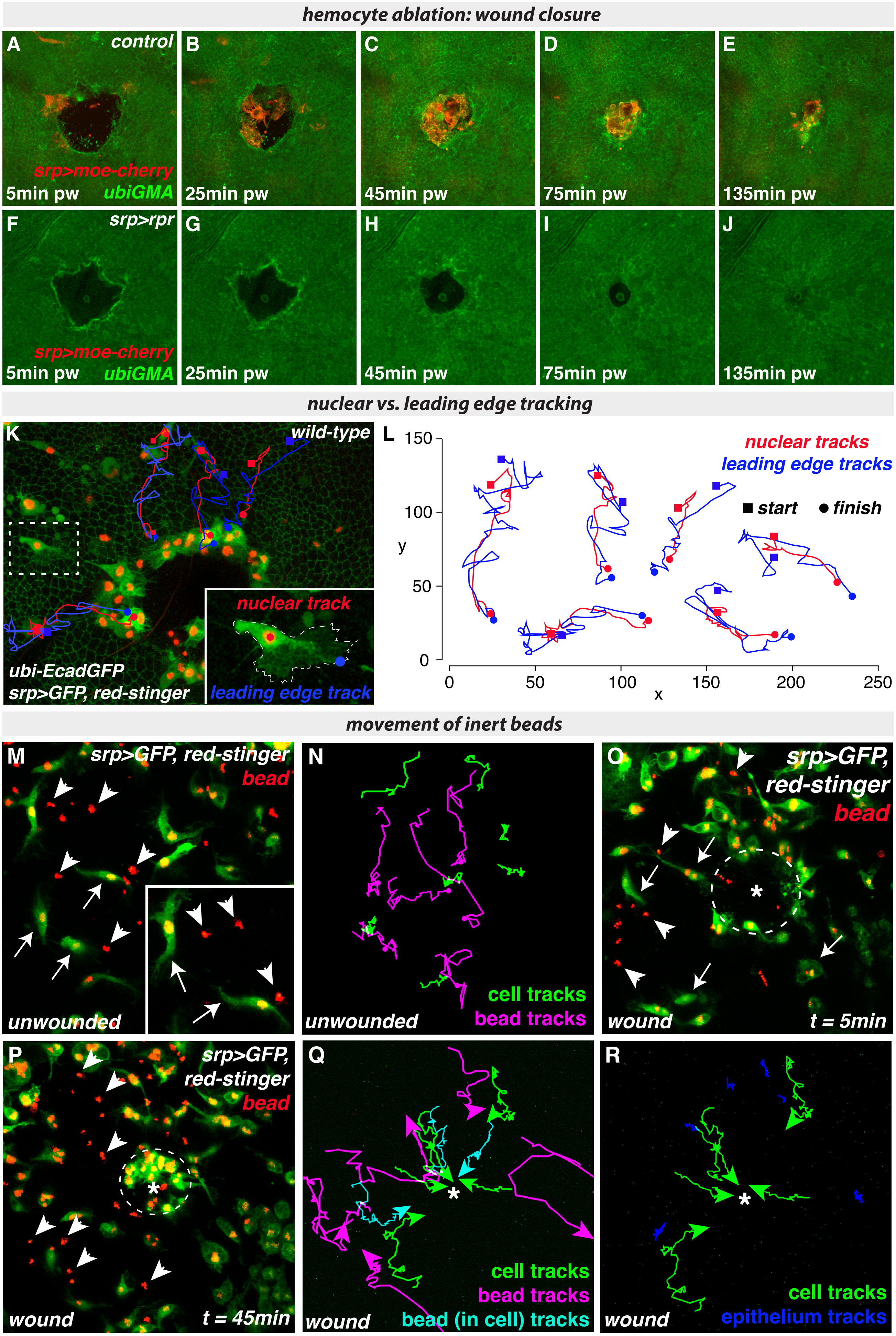
hemocyte ablation: wound closure
A control
srp>moe-cherry
5min pw ubiGMA
B 25min pw
C 45min pw
D 75min pw
E 135min pw
F srp>rpr
srp>moe-cherry
5min pw ubiGMA
G 25min pw
H 45min pw
I 75min pw
J 135min pw
nuclear vs. leading edge tracking
K wild-type
ubi-EcadGFP
srp>GFP, red-stinger
nuclear track
leading edge track
L
nuclear tracks
leading edge tracks
start
finish
y
x
movement of inert beads
M srp>GFP, red-stinger
bead
unwounded
N
cell tracks
bead tracks
unwounded
O srp>GFP, red-stinger
bead
wound
t = 5min
P srp>GFP, red-stinger
bead
wound
t = 45min
Q
cell tracks
bead tracks
wound
bead (in cell) tracks
R
cell tracks
epithelium tracks
wound

Figure S2

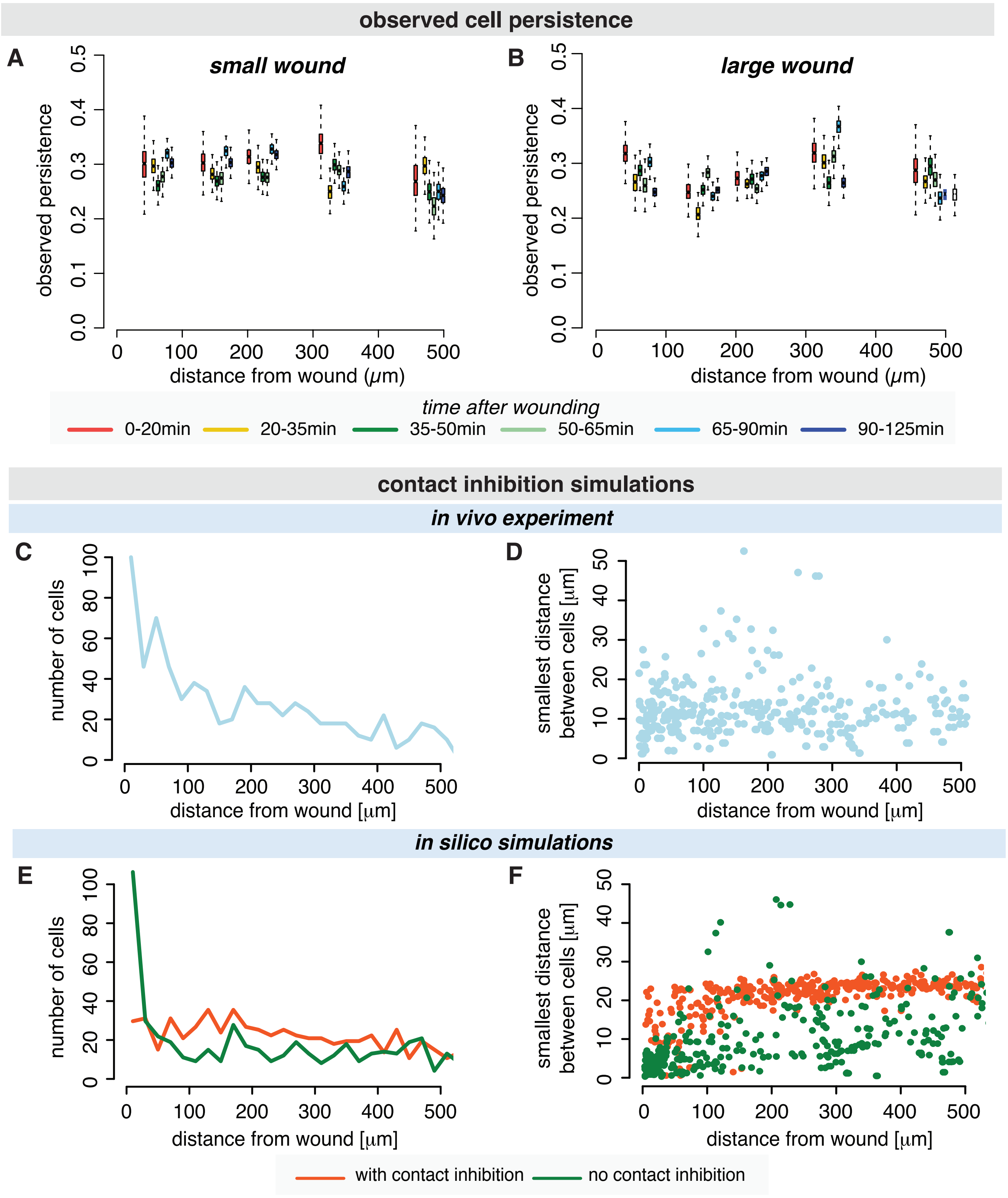

observed cell persistence
A
small wound
observed persistence
0.0 0.1 0.2 0.3 0.4 0.5
0 100 200 300 400 500
distance from wound (μm)
B
large wound
observed persistence
0.0 0.1 0.2 0.3 0.4 0.5
0 100 200 300 400 500
distance from wound (μm)
time after wounding
0-20min
20-35min
35-50min
50-65min
65-90min
90-125min
contact inhibition simulations
in vivo experiment
C
number of cells
0 20 40 60 80 100
0 100 200 300 400 500
distance from wound [μm]
D
smallest distance between cells [μm]
0 10 20 30 40 50
0 100 200 300 400 500
distance from wound [μm]
in silico simulations
E
number of cells
0 20 40 60 80 100
0 100 200 300 400 500
distance from wound [μm]
F
smallest distance between cells [μm]
0 10 20 30 40 50
0 100 200 300 400 500
distance from wound [μm]
with contact inhibition
no contact inhibition

Figure S3

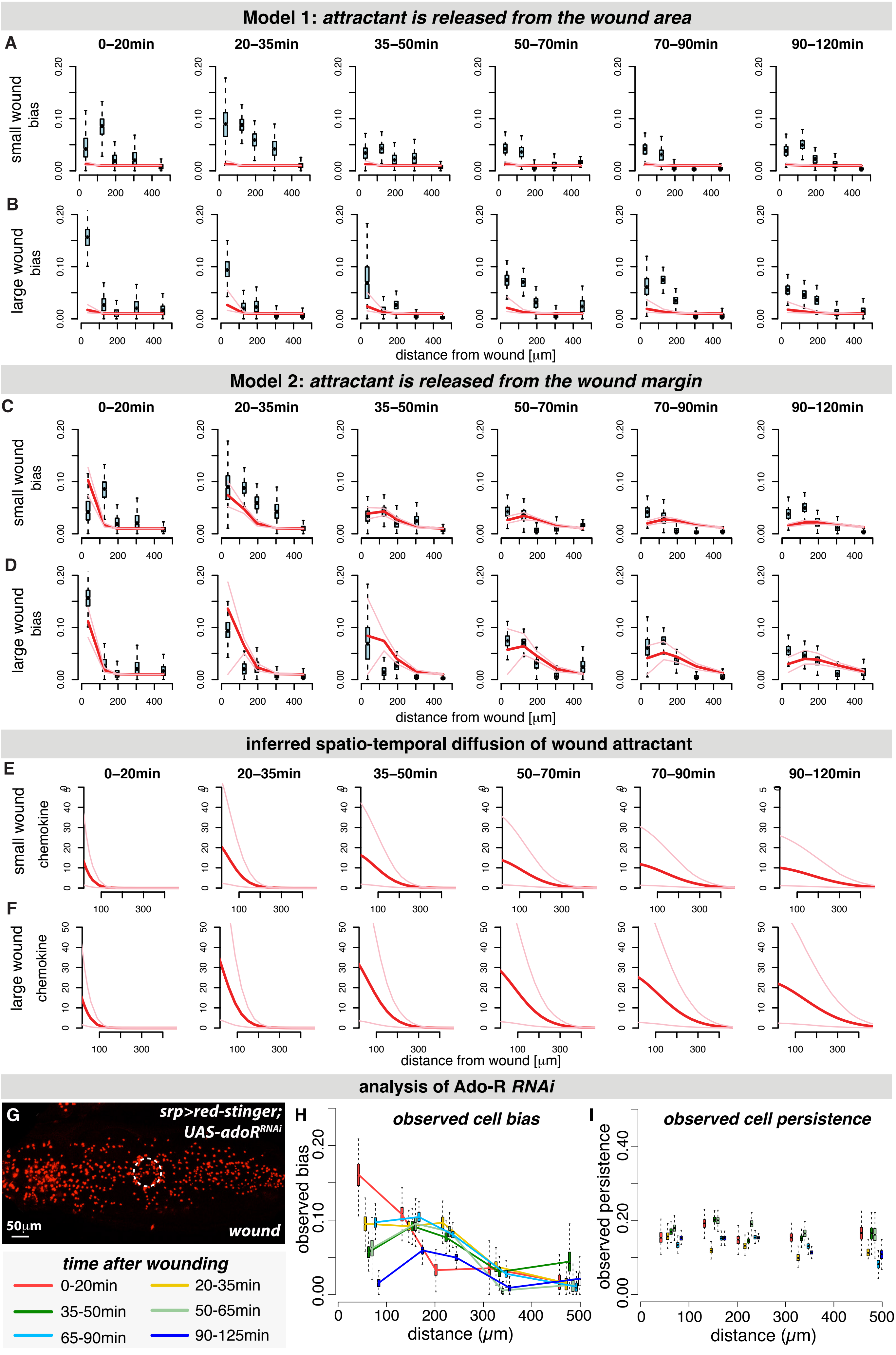

Model 1: attractant is released from the wound area
A
B
0–20min
20–35min
35–50min
50–70min
70–90min
90–120min
small wound bias
large wound bias
distance from wound [μm]
Model 2: attractant is released from the wound margin
C
D
inferred spatio-temporal diffusion of wound attractant
E
F
small wound chemokine
large wound chemokine
analysis of Ado-R RNAi
G
srp>red-stinger; UAS-adoR^RNAi
50μm
wound
H
observed cell bias
observed bias
distance (μm)
I
observed cell persistence
observed persistence
time after wounding
0-20min
20-35min
35-50min
50-65min
65-90min
90-125min

Figure S4

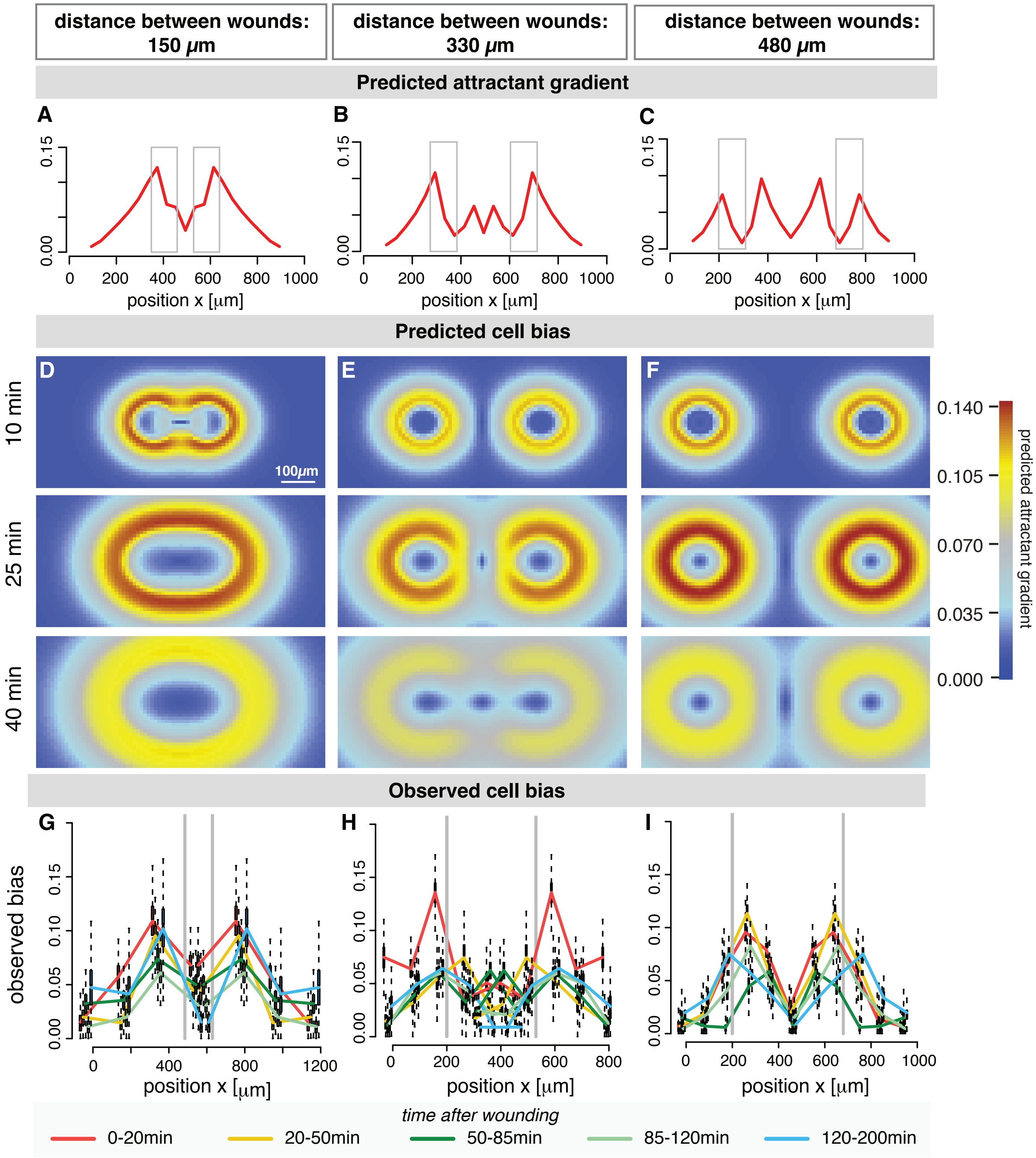

distance between wounds: 150 μm
distance between wounds: 330 μm
distance between wounds: 480 μm
Predicted attractant gradient
A
B
C
0.00
0.15
0
200
400
600
800
1000
position x [μm]
Predicted cell bias
D
E
F
10 min
25 min
40 min
100μm
0.140
0.105
0.070
0.035
0.000
predicted attractant gradient
Observed cell bias
G
H
I
observed bias
0.00
0.05
0.10
0.15
0
400
800
1200
200
600
1000
position x [μm]
time after wounding
0-20min
20-50min
50-85min
85-120min
120-200min

Figure S5

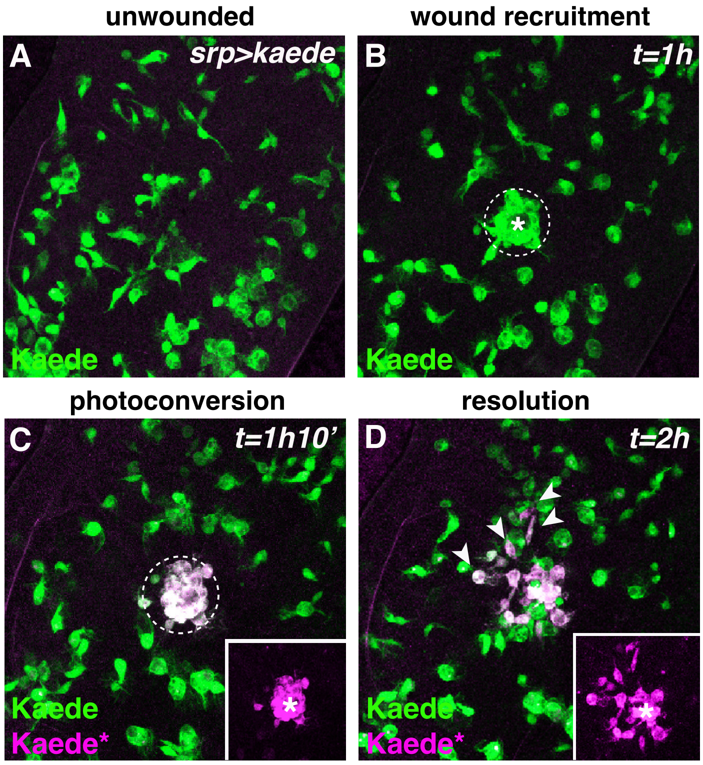

unwounded
wound recruitment
A
srp>kaede
B
t=1h
Kaede
Kaede
photoconversion
resolution
C
t=1h10'
D
t=2h
Kaede
Kaede*
Kaede
Kaede*

Figure S6

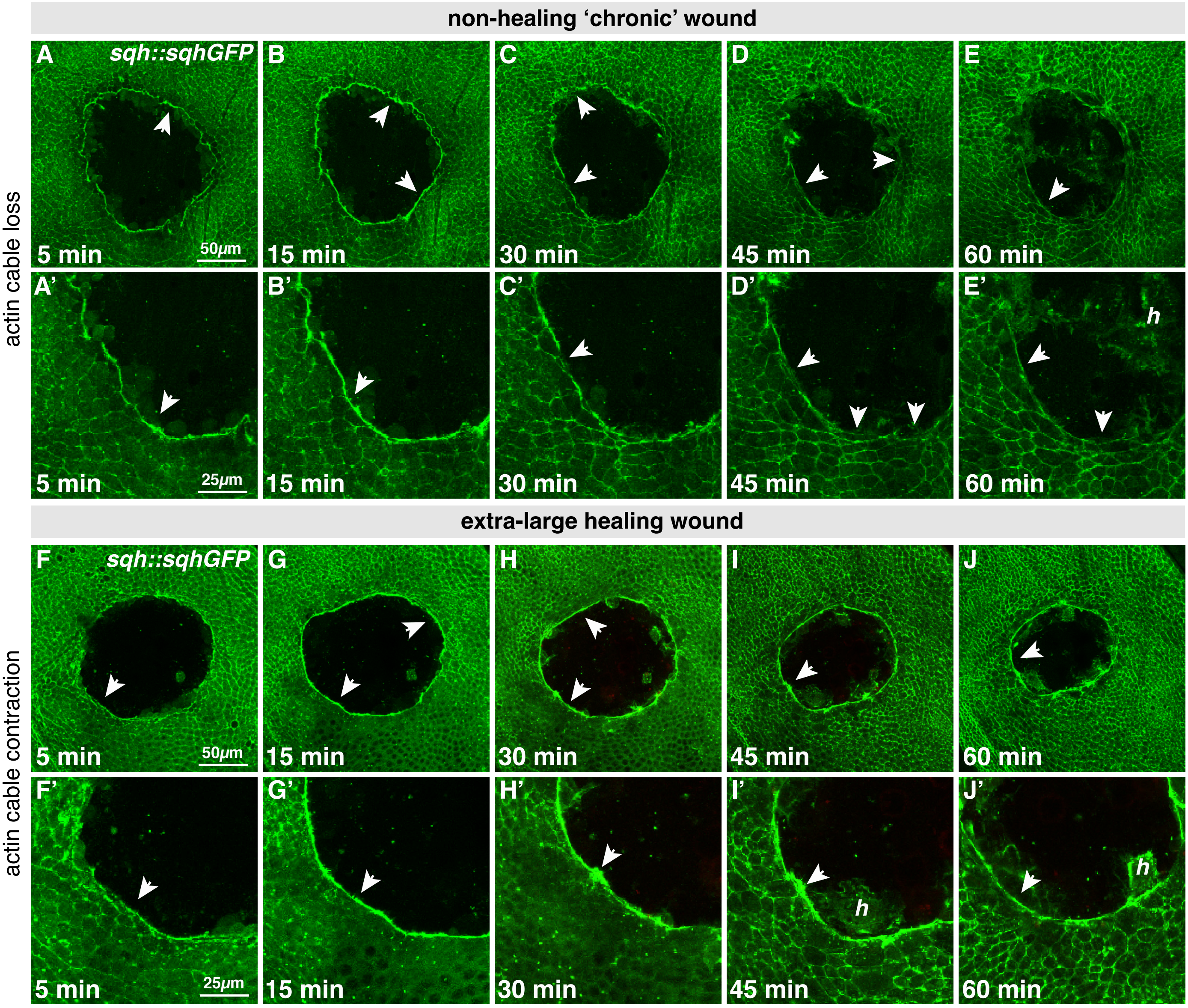

non-healing 'chronic' wound
actin cable loss
A
sqh::sqhGFP
B
C
D
E
5 min
50µm
15 min
30 min
45 min
60 min
A'
B'
C'
D'
E'
h
5 min
25µm
15 min
30 min
45 min
60 min
extra-large healing wound
actin cable contraction
F
sqh::sqhGFP
G
H
I
J
5 min
50µm
15 min
30 min
45 min
60 min
F'
G'
H'
I'
J'
h
h
5 min
25µm
15 min
30 min
45 min
60 min

Supplemental Figure Legends

Figure S1. Related to Figure 1. Hemocytes are not required for pupal wound closure and hemocyte movement is not driven by bulk tissue flow

(A-J) Wound closure in control samples (A-E) is accompanied by hemocyte recruitment (red, *srp-Gal4* driven *mCherry-Moesin*) but hemocyte ablation (*srp-Gal4* driven expression of pro-apopotic gene *reaper*) does not impair wound closure (F-J). Wounded epithelium labelled using ubiquitously-expressed *GFP-tagged Moesin* (green).

(K-L) Nuclear tracking of hemocytes correlates well with the dynamic behaviour of the hemocyte leading edge during recruitment to the wound site. Example nuclear tracks shown in red and corresponding leading edge tracks shown in blue. Squares represent the start of the hemocyte track and circles represent the final tracked position.

(M-R) Co-injection of inert fluorescent beads (red, arrowheads) into the extracellular space of pupal wings. Hemocytes (arrows) labelled by *srp-Gal4* driven expression of cytoplasmic GFP (green) and nuclear RFP (red). Beads move rapidly and randomly in the extracellular space of unwounded wings (majenta, N) compared to the slower moving hemocytes (green, N). Following wounding, hemocytes migrate directionally towards the injury (green, Q and R) but extracellular beads are not biased towards the wound (majenta, Q). Beads engulfed by hemocytes (cyan, Q) move towards the wound in a similar way to hemocytes without beads (green, Q). There is minimal movement of the overlying epithelium towards the wound, shown by the negligible movement of beads that have been engulfed by epithelial cells (blue, R) compared to the hemocytes (green, R).

Figure S2. Related to Figure 2. Hemocytes do not change migration persistence upon wounding and do not undergo contact-inhibition of locomotion at wounds

(A-B) The observed cell persistence, extracted from *in vivo* hemocyte trajectory data, is not changed upon wounding for either small (A) or large (B) wounds and remains at baseline levels observed in control unwounded samples. Each boxplot represents a marginal posterior parameter distribution for the observed persistence estimated for each spatio-temporal cluster.

(C-F) The number of hemocytes found at varying distances from the wound site as observed in vivo (C) or predicted from in silico simulations (E) with hemocytes either undergoing contact inhibition of locomotion (red) or not (green). Also plotted is the smallest distance found between hemocytes at different locations from the wound site as observed in vivo (D) or predicted from in silico simulations (F). In silico simulations based on hemocytes migrating without contact inhibition (green, E and F) best fit the experimental data (C and D).

Figure S3. Related to Figure 3. Calibrating the model of the diffusion gradient to infer parameters of the attractant gradient.

(A-D) Determining which model of the diffusion gradient best-fits the observed *in vivo* data. The attractant is either released from the wound area i.e. the damaged necrotic tissue (A and B) or the attractant is released from cells around the wound margin (C and D). The observed cell bias from *in vivo* data (blue box plots) is compared with that of the expected bias according to the different models (red lines). The model that fits the experimental data is determined to be the one in which the attractant emanates from the wound margin.

(E-F) The inferred spatio-temporal diffusion gradient of the wound attractant, according to the model in which the attractant emanates from the wound edge, at different times and distances post-wounding for small (E) and large (F) wounds. Red lines indicate the mean and pink lines indicate the 5%- and 95%-tile of 1000 simulations from the inferred posterior distributions.

(G-I) RNAi-mediated knockdown of the *Drosophila* ATP receptor AdoR specifically in hemocytes using the *srp-Gal4* driver (G) does not impair the hemocyte response to wounding (cell bias in H and cell persistence in I).

Figure S4. Related to Figure 4. Modelling competing attractant gradient interactions in response to double wounds.
(A-C) For wounds close together, attractant gradients overlap and mimic a single very large wound, predicting less biased migration in the inter-wound region (red line, A). For wounds far apart, attractant gradients will not interact and hemocytes respond as for two single wounds (red line, C). For wounds of intermediate distance apart, attractant gradients will strongly overlap, creating shallower gradients and reduced bias in the inter-wound region (red line, B). Grey boxes indicate wound positions.
(D-F) Modelling predicts the resultant cell bias following the spatial interaction of competing gradients from two adjacent wounds. For two wounds that are positioned close together, the attractant gradients will rapidly merge, mimicking that of a single very large wound (A). For wounds that are far apart, the two wounds will behave separately and cells will respond similar as for two independent individual wounds (C). For wounds at an intermediate distance apart, the attractant gradients will interact in the inter-wound region after 25min post-wounding, causing a drop in bias in this region (B).
(G-I) The observed cell bias extracted from *in vivo* imaging data obtained for each of these three different scenarios is consistent with the *in silico* predictions and confirms the validity of the inferred attractant gradient parameters for the single wound. Each boxplot represents a marginal posterior parameter distribution for the observed bias estimated for each spatio-temporal cluster.

Figure S5. Related to Figure 5. Photoconvertible fluorophore Kaede permits selective labelling and tracking of wound-recruited hemocytes.
(A-D) Photoconvertible fluorophore Kaede expressed specifically in hemocytes using the *srp-Gal4* driver (green, A). Wounding (asterisk) induces the recruitment of hemocytes to the injury site (dotted circle, B) which are selectively labelled by UV-mediated photoconversion of Kaede in these cells (majenta, C; also see inset). Photoconverted cells (Kaede*, majenta) can then be tracked as hemocytes resolve away from the wound (arrowheads, D; also see inset).

Figure S6. Related to Figure 6. Non-healing chronic wounds do not assemble a stable actomyosin cable at the leading edge.
(A-J) Actomyosin dynamics during wound closure visualised using GFP-tagged *sqh* (the *Drosophila* regulatory light chain of non-muscle myosin). Unlike normal healing wounds that assemble a stable contractile actomyosin cable at the leading edge (arrowheads, F-J) that facilitates wound closure, non-healing ‘chronic’ wounds lose the actomyosin cable (arrowheads, D-E) that initially assembles (arrowheads, A-C).

Supplemental Experimental Procedures

Image processing and cell tracking

Imaging resulted in image stacks with dark background and red fluorescent cell nuclei. All image processing and cell tracking algorithms were implemented in *R* using the package EBImage [S1]. The information of the cell nuclei was extracted automatically from the images using an edge detection method. A manually set threshold of the light intensity was used per image stack. Each detected cell nucleus was described as an object with the coordinates of its geometrical center indicating the cell nucleus location and the time the cell was observed. The cell nuclei were tracked and reconstructed over the z-stack using a surface algorithm. The surface algorithm was then applied to track reconstructed cells over time, which is based on the shortest distance between cells from two consecutive images. We excluded all cell trajectories that included time points in which the cell was located at the edge of the image. For each image stack the extracted cell tracks were checked and curated manually. The images included cell tracks of the wing disc and other cell tracks, resulting from leukocytes migrating in other parts of the pupa. All cell tracks outside of the wing disc were discarded manually. Each cell track was stored as an *R* object, containing its 3D coordinates, its corresponding time points after wounding and the location of the wound(s). Cell tracks from image stacks taken under the same experimental conditions were merged into a single data set and stored as *RData* files for further analysis.

Leukocyte migration model

We apply a biased persistent random walk model [S2, S3] for our leukocyte migration model. The model consists of N non-interacting leukocytes (particles), represented as 2 dimensional coordinates in the wing disc. The direction of a cell's movement at any time step t is described by two random variables, which are a step length s_t and a turning angle γ_t. Since our statistical analysis is based purely on directional statistics (as opposed to distance measures such as mean-squared displacement), there is no restriction on the distribution of step lengths to be used in the model. Nevertheless for purely practical purposes, we model the step lengths according to $s_t \sim \sqrt{dt}\,\mathrm{N}^+(0,1)$, where $N^+(0, 1)$ is a truncated standard normal distribution with support on the positive real axis, and $dt = 0.001$. The turning angle γ_t is defined without loss of generality as the angle between a motion vector and an arbitrary reference axis, which we take here to be the negative y-axis (see Fig. 1a), at time *t*. γ_t follows the wrapped normal distributions [S4] with the probability density function

$$N_w(\gamma_t|\mu,\sigma) = \frac{1}{\sigma\sqrt{2\pi}} \sum_{i=-\infty}^{\infty} \exp\left(-\frac{(\gamma_t-\mu+2\pi i)^2}{2\sigma^2}\right).$$

The mean μ and variance σ depend on whether the random walker follows a biased or persistent motion. For the biased motion we define $\mu = \beta$ (the direction of bias, *i.e.* the vector that points from the cell center towards the center of the wound) and for the persistent distribution we have $\mu = \gamma_{t-1}$, which is the direction in the previous time step. The variances σ for the biased and persistent motion are denoted as $\sigma_p = -2\log(p)$ and $\sigma_b = -2\log(b)$, respectively, with the persistence and bias parameters, p and b taking values between 0 and 1. They affect the variance of the distributions such that the closer to 1 they are, the smaller their respective variances will be, and the more likely the particle will be to sample an angle in the direction of the bias or the persistence, meaning a cell has a stronger level of bias or persistence. If p or b is equal to 0, then the corresponding variance will tend to infinity and thus the distribution is a wrapped uniform distribution, meaning the cell will not exhibit any bias or persistence.

The decision whether the cell follows biased or persistent motion is based on a further random variable, which follows a Bernoulli distribution with success probability *w*, so that *w* describes the probability of a biased motion and *1-w* describes the probability of a persistent motion. At each time point, γ_t and s_t are determined and, accordingly, the cell moves a distance of s_t in the direction defined by γ_t. This constitutes one step in the cell's trajectory, described by the 3 parameters (*p*, *b* and *w*).

Inference of observed bias and persistence

From our observed leukocyte trajectory data, we infer the parameters of the bias persistent random walk model in the framework of Bayesian statistics. The likelihood *L(b, p, w)* function can be derived exactly [S2] and we sampled the joint posterior distribution of the three parameters using a standard Markov Chain Monte Carlo sampler. The computation was implemented in *R* and was run over 5000 MCMC steps with the first 3000 discarded as burn-in. We used an adaptive log-Gaussian kernel for each parameter separately with the variance equal to half the variance of all previously sampled parameter values. The posterior parameter distributions for b, p and w are summarized into the *observed bias (=w*b)* and the *observed persistence (=(1-w)*p)*. The inference scheme was applied to each spatio-temporal cluster separately and the summarized marginal posteriors are shown in the figures in this paper as boxplots.

Attractant diffusion model

In absence of any a priori information about the attractant characteristics we make use of the simplest diffusion model, i.e. the standard diffusion equation in two dimensions. Attractant *a* is produced with strength *A* from time $t = 0$ till $t = \tau$, with the source at position $x = x_0$. To obtain the attractant concentration we then have

$$a(x,t) = \int_0^{\min(t,\tau)} \frac{A}{\sqrt{4D(t-\varsigma)\pi}} \exp\left(-\frac{(x-x_0)^2}{4D(t-\varsigma)}\right) d\varsigma,$$

where *D* is the diffusion coefficient. Two models for the attractant source were investigated. Model 1: All epithelial cells that are part of the wound area produce the attractant. Each of these cells will act as a source, uniformly distributed on the wound area. Model 2: Only the epithelial cells that are located at the wound margin produce the attractant. Each of these cells will act as a source, uniformly distributed on the wound margin. In both models the radius of epithelial cell was set to 5 μm. The number of epithelial cells was proportional to the wound area (model 1) and wound circumference (model 2). The diffusion equation was solved for each emitting cell, and the total attractant concentration was determined.

Solving the attractant diffusion models (using numeric integration) we can then compute the local attractant diffusion gradient for each point *x* in the wing disc and at each time *t* of interest. We set the average radius of a leukocyte to r = 15μm. A leukocyte is sensing the local attractant concentration at its front (site that is closest to the wound) and at its rear (site furthest away from the wound). Therefore, for a leukocyte at position x_t we need to determine the attractant concentration $a_f = x_t - r$ and $a_r = x_t + r$. We apply standard receptor-ligand binding kinetics for attractant sensing, assuming that the steady state for the receptor-attractant complex is on a much faster time scale than the resulting movement of the leukocyte. The signal a leukocyte is sensing is then proportional to the concentration of receptor-attractant–complex at its front $[Ra_f]$ and rear $[Ra_r]$:

$$[Ra_f] = \frac{1}{2}(K_d + R_0 + a_f) - \sqrt{\frac{1}{4}(K_d + R_0 + a_f)^2 - R_0 a_f}$$

and

$$[Ra_r] = \frac{1}{2}(K_d + R_0 + a_r) - \sqrt{\frac{1}{4}(K_d + R_0 + a_r)^2 - R_0 a_r}\,,$$

where R_0 is the total local receptor concentration and K_d is the dissociation constant. Since we do not observe any changes in the observed persistence upon wounding, we assume that only the leukocyte's observed bias depends on the attractant gradient. We do not have any information about the nature of the attractant and the resulting intracellular signaling cascades that are triggered by the attractant and that result into translation of the signal to actual leukocyte movement, we propose that the cells movement (strength of observed bias) depends on the difference of sensed attractant in a linear relationship:

$$ob = m([Ra_f] - [Ra_r]) + b_0,$$

where ob is the observed bias, m is a scaling constant and b_0 is the baseline observed bias, which was extracted from leukocytes in unwounded wing discs.

Inference of attractant dynamics

The model parameters of the attractant diffusion model (D, A, τ, m, R_0 and K_d) were estimated using the determined marginal posterior distributions for the observed biased of each spatio-temporal cluster simultaneously for small and large wounds. All parameters were assumed to be the same for small and large wounds, except τ, which was estimated as t_s and t_l for small and large wounds, respectively. We apply the same inference scheme as above. The likelihood for the parameter set θ is then simply defined as:

$$L(\theta) = \prod_{i=1}^{T} \prod_{j=1}^{S} p(ob_{i,j}|\theta),$$

where T is the number of temporal clusters, S is the number of spatial clusters and $p(ob_i|\theta)$ is the probability density estimated from the marginal posterior distribution of the observed bias for each spatio-temporal cluster, respectively.

The MCMC scheme was run over 10^6 particles, with 500,000 particles discarded as burn-in. All remaining algorithm parameters were set as above. We apply the same scheme to both attractant gradient models, resulting in reasonable model fits only for model 2 (attractant is released from the wound margin). We repeated the inference scheme for both models 10 times with random starting values to (i) ensure as much as possible that the algorithm converges to the same posterior distributions (ii) to avoid the estimation of local minima.

***In silico* predictions and attractant diffusion model accounting for wing geometry**

All simulations were done 100 times with parameters sampled from the posterior distributions obtained from single wound data. Shown throughout this paper are the mean of the simulation results (in heat maps) or the mean and the 95 and 5 percentiles of the simulation results (as trajectories).

To simulate the diffusion of attractant under the consideration of the wing disc geometry, we solved numerically the standard diffusion equation in 2D [S5] for a rectangular area of 1000μm x 200μm, representing the wing disc. The scheme was implemented in R.

***In silico* contact inhibition model**

We adapted our leukocyte migration model to account for contact inhibition. We applied the mathematical description for contact inhibition as described in [S6]. 300 leukocyte trajectories in response to a single wound were simulated on the drosophila wing disc (approximated by a rectangular

area of 1000μm x 200μm). To compare the simulation results with the *in silico* leukocyte migration model in absence of contact inhibition and to the trajectory data extracted from our imaging data sets, we computed how many cells are located at a given distance from wound as well as the smallest distance between cells. The *in silico* simulations of the leukocyte migration model in absence of contact inhibition are in agreement with the experimental data.

Methodological Primer
Mathematical and computational concepts behind the modelling approaches

Background

Mathematical and computational modelling has become an important tool in modern biology. In particular, it enables us to capture relationships and processes that are not necessarily directly accessible by current experimental techniques; in this sense mathematical modelling complements and extends the scope of experimental approaches.

An integrated experimental-modelling approach can therefore elucidate even complex molecular processes. But like their experimental counterparts, the mathematical tools need to be tailored to the problem at hand, and a variety of approaches have been developed that can be used to model different biological systems. Importantly, at the outset both modeller and biologist have to state their assumptions concisely and unambiguously.

The basic procedure to integrate modelling and experiment follows a generic pattern. In order to combine experimental analysis with modelling we require tools to:

(i) calibrate (or "fit") models to data;
(ii) test whether the fit is satisfactory;
(iii) formally select between different competing models or hypotheses;
(iv) extract testable and non-trivial predictions from the analysis of the calibrated model(s);
(v) extend and further develop the model used to describe the biological system in an iterative (back and forth) manner.

Steps (iv) and (v), more than any of the others, require considerable amounts of expertise to guide the development and further refining of mathematical models (see schematic below). In the following we will briefly outline the mathematical and computational concepts applied in this study for each of the above-mentioned points.

Model calibration

The majority of mathematical or computational models that describe dynamical systems contain parameters. These parameters can be reaction rate constants (e.g. the rate constant of protein degradation), physical parameters such as the volume of a cell or the rate of cell division, or maybe more abstract parameters that do not have a direct obvious relationship to the biophysical processes going on in the biological system but provide useful descriptions of parts of these systems that would otherwise be hard to model in detail. Although model parameters are often unknown, they are needed in order to learn about the model characteristics and to make useful model predictions.

Some parameters can be obtained experimentally, but for many parameters, especially for *in vivo* studies, this may not be feasible. One possibility is to 'borrow' parameters from the literature. However, this approach is often not suitable, because literature parameter values may have been obtained under experimental conditions that differ to the experiments of interest. The aim of model calibration is to link the mathematical model with observed experimental data in order to determine the model parameters.

In our study we calibrate two models: (i) a model of hemocyte migration and (ii) a model of gradient dynamics. For simplicity we will focus here on the first model (i). This model contains three

parameters, describing a cell's bias, the cell's persistence and a 'weighting parameter' (weighting here refers to the balance between bias and persistence). We need to determine these three parameters for every spatio-temporal cluster and for each treatment group (unwounded controls, small/large/extra-large single wounds and double wounds, made at the same or different times).

Model calibration can be done in several ways. Here, we employ a Bayesian statistical framework to infer the model parameters from experimental data. The overall idea is to combine our prior knowledge of the behaviour of the cells with the likelihood function, which defines the probability of observing the experimental data from the model with a specific set of parameters. The parameter set with the greatest statistical confidence (technically the maximum a posteriori probability (MAP) estimate) is then considered the true parameter set. In informal terms: we take our mathematical model, plug in thousands of potential parameter combinations and determine which model output fits best to our experimental data and prior knowledge. The result is a 'posterior parameter distribution' which describes not only each parameter separately, but also contains the relationship between the estimated parameters. The 'marginal posterior parameter distributions' provide a subset of the entire posterior distribution, but ignores the relationships to the remaining parameters. One thing to note: This approach does not provide a single parameter set; instead it provides a distribution over all possible parameter sets, which provides the best possible confidence estimates for each parameter. Crucially, this framework focuses on achieving practical significance of results; which experience shows, is a more useful (but harder to achieve) objective than merely statistical significance.

Coming back to our cell migration model: We estimate all three parameters for each spatio-temporal cluster. The resulting (posterior) parameter distribution, which consists of the three parameters, can then be summarised into two parameters (observed bias and observed persistence) with appropriate weighting. We then look at the distributions of the summarised parameters, which we represent across the manuscript as boxplots. This means that every boxplot shown represents the estimated distribution of a model parameter based on the experimental imaging data.

<u>Model selection</u>

Researchers often find themselves in situations, where they can define a whole set of models, rather than a single one, that all describe the same biological process. This brings us to the field of model selection.

To illustrate this, assume we have two potential mathematical models, *model A* and *model B*, aiming to describe the same biological process, e.g. migration pattern of hemocytes. We want to understand, which model has the higher probability to represent our data best. Again, we can use the Bayesian inference framework to distinguish between the two models. Before doing so, we must consider an important issue: The two candidates, model A and model B, both contain a set of parameters, but their complexity may be very different. *Model A* might summarise all processes related to the intracellular signalling and resulting migration of cells into a single equation, while *model B* describes the details of receptor signalling, intracellular signalling processes, biophysical changes of the cytoskeleton and the resulting hemocyte migration. Both models might be useful depending on the question we aim to answer and depending on available experimental data. However, the more important point in the model selection framework is that *model A* will have far fewer model parameters than *model B*. One can almost *always* improve a model's fit to experimental data by including more parameters in a model, but this does not imply that the model is a better representation of the physical system. Any statistical framework for model selection should take this into account, i.e. it should 'penalise more complex models compared to less complex models.

In this study we present two model selection problems: (i) the attractant is emitted by the wound area vs. the attractant is emitted by the wound margin; and (ii) there is a single wave of attractant vs. there are two waves of attractant. We will now discuss both scenarios, because they highlight two different aspects of model selection.

1. The attractant is emitted by the wound area (model 1) vs. the attractant source is emitted by the wound margin (model 2).

We first try to obtain model fits for both models. In this case, we were not able to obtain any reasonable fits to the data for *model 1*, while the fits for *model 2* were satisfactory. In this simple scenario we can reject *model 1* and consider *model 2* as the better model to explain the data.

2. There is a single wave of attractant (model 1) vs. there are two waves of attractant (model 2).

Again, we first try to obtain model fits for both models. In this case both models fit the data well, but *model 2* fits the data slightly better. Note, *model 1* is less complex than *model 2*, because *model 2* contains additional parameters describing the second wave of attractant (resulting in nearly twice as many model parameters compared to *model 1*). We then had to investigate, if the slightly better fit of model 2 justifies the larger number of parameters; this is a classic example of model selection, where we have to balance the complexity of a model with its ability to reflect the data. There are several ways to do so and here we were able to use one of the simplest, the so-called *likelihood ratio* statistical test. This test computes how much more likely *model 2* explains the data than *model 1*. Applying this test we found that we can not reject the null hypothesis (*model 2* and *model 1* one can equally well explain the data). How should this be interpreted in the biological context? We find that a single attractant wave can best explain our experimental data and conclude that there is no evidence that there is a second wave of attractant that recruits hemocytes to the wound site.

***In silico* predictions**

Once a model is constructed and calibrated it can be used for *in silico* predictions and simulations. Such predictions can serve several purposes, including model validation and actual predictions of the outcomes of experiments that might or might not be doable in practice. Model validation is an important part of model development and should be done whenever possible. In our study, the mathematical model is used to predict the outcome of a new experiment, which can then be performed in practice. If the model is useful, its prediction should match with the newly performed experiment. We note, that simulations from the model, and the comparison of the simulation results with the data (including the original data used to calibrate the model(s)) should be a crucial first step in any assessment of the model's relevance or validity.

In our study, we predicted the response of the hemocytes in response to two wounds occurring at the same time but at a distance apart, which we then experimentally verify (Figure 4); this served as important validation of our model. We also aimed to predict the response of hemocytes to a second wound that was induced a certain time after a first wound. In this latter case, our model fails to predict the correct hemocyte behaviour but instead uncovered an interesting biological phenomenon (Figure 5). The model is still valid for the majority of the aspects investigated in this study, but we have identified the limits of the model. More specifically, the model only includes basic aspects of hemocyte migration; it cannot include behaviours that we do not yet know. For example, we did not include the phenomenon of desensitisation of hemocytes which is why our simulation did not mirror the actual biology, and it was this difference that revealed the new phenomenon and encouraged us to investigate further i.e. to determine how long the hemocytes remain desensitised to a second wound.

An important aspect of *in silico* predictions is uncertainty. As described above, we need to use our estimated model parameters to obtain *in silico* predictions. But how reliable are the predictions? What are the confidence intervals? In our model calibration framework we obtain parameter distributions, rather than single parameter values. We now have a semi-realistic generative model where these distributions can be used to simulate the physical system to generate *in silico* predictions. Rather than simulating the model with a single parameter value, we perform multiple simulations, approximately 1000 iterations, each time with a different parameter set drawn from our estimated parameter distribution. We now have 1000 model predictions, from which we can compute the mean, i.e. the most likely behaviour and the accompanying confidence intervals.

Schematic of the model development pipeline. *In vitro* experiments and literature can be used to generate initial hypotheses, which are then analysed and tested with computational methods. Once good agreement between the mathematical model and the experimental data is found, the model is validated on further experiments. The final model can be used for *in silico* predictions and experimental design.

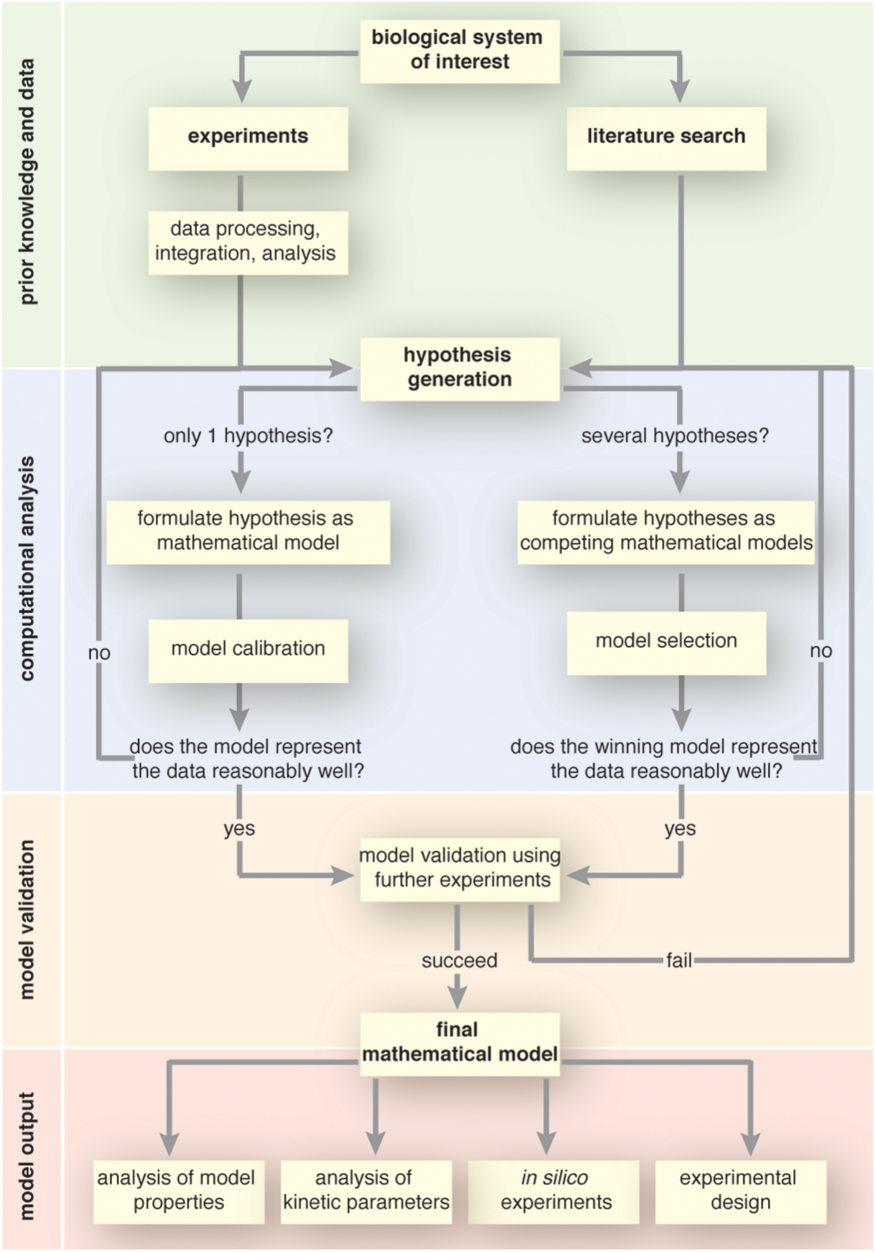

Supplemental References

S1. Pau, G., Fuchs, F., Sklyar, O., Boutros, M., and Huber, W. (2010). EBImage--an R package for image processing with applications to cellular phenotypes. Bioinformatics *26*, 979–981.

S2. Jones, P. J. M., Sim, A., Taylor, H. B., Bugeon, L., Dallman, M. J., Pereira, B., Stumpf, M. P. H., and Liepe, J. (2015). Inference of random walk models to describe leukocyte migration. Phys. Biol. *12*, 066001.

S3. Liepe, J., Taylor, H., Barnes, C. P., Huvet, M., Bugeon, L., Thorne, T., Lamb, J. R., Dallman, M. J., and Stumpf, M. P. H. (2012). Calibrating spatio-temporal models of leukocyte dynamics against in vivo live-imaging data using approximate Bayesian computation. Integr. Biol. (Camb). *4*, 335–45.

S4. Breitenberger, E. (1963). Analogues of the Normal Distribution on the Circle and the Sphere. Biometrika *50*, 81.

S5. Rossant, C. (2014). IPython Interactive Computing and Visualization Cookbook (Packt Publishing Ltd)

S6. Davis, J. R., Huang, C.-Y., Zanet, J., Harrison, S., Rosten, E., Cox, S., Soong, D. Y., Dunn, G. a, and Stramer, B. M. (2012). Emergence of embryonic pattern through contact inhibition of locomotion. Development *139*, 4555–60.
